# A kinase-independent role for CDK8 in BCR-ABL1^+^ leukemia

**DOI:** 10.1038/s41467-019-12656-x

**Published:** 2019-10-18

**Authors:** Ingeborg Menzl, Tinghu Zhang, Angelika Berger-Becvar, Reinhard Grausenburger, Gerwin Heller, Michaela Prchal-Murphy, Leo Edlinger, Vanessa M. Knab, Iris Z. Uras, Eva Grundschober, Karin Bauer, Mareike Roth, Anna Skucha, Yao Liu, John M. Hatcher, Yanke Liang, Nicholas P. Kwiatkowski, Daniela Fux, Andrea Hoelbl-Kovacic, Stefan Kubicek, Junia V. Melo, Peter Valent, Thomas Weichhart, Florian Grebien, Johannes Zuber, Nathanael S. Gray, Veronika Sexl

**Affiliations:** 1 Institute of Pharmacology and Toxicology, University of Veterinary Medicine, Vienna, Austria; 2 Department of Cancer Biology, Department of Biological Chemistry and Molecular Pharmacology, Dana-Farber Cancer Institute, Harvard Medical School, Boston, Massachusetts USA; 3 Department of Medicine I, Medical University of Vienna, Vienna, Austria; 4 Comprehensive Cancer Center, Vienna, Austria; 5 Division of Hematology and Hemostaseology, Department of Internal Medicine I, Ludwig Boltzmann Institute for Hematology and Oncology, Medical University of Vienna, Vienna, Austria; 6 Research Institute of Molecular Pathology, Campus-Vienna-Biocenter 1, Vienna, Austria; 7 Ludwig Boltzmann Institute for Cancer Research, Vienna, Austria; 8 Research Center for Molecular Medicine of the Austrian Academy of Sciences, Vienna, Austria; 9 Faculty of Health and Medical Sciences, University of Adelaide, Adelaide, South Australia 5005 Australia; 10 Department of Hematology, Imperial College London, Kensington, London, SW7 2AZ UK; 11 Center of Pathobiochemistry and Genetics, Institute of Medical Genetics, Medical University of Vienna, Vienna, Austria; 12 Institute for Medical Biochemistry, University of Veterinary Medicine, Vienna, Austria

**Keywords:** Cancer therapy, Haematological cancer

## Abstract

Cyclin-dependent kinases (CDKs) are frequently deregulated in cancer and represent promising drug targets. We provide evidence that CDK8 has a key role in B-ALL. Loss of CDK8 in leukemia mouse models significantly enhances disease latency and prevents disease maintenance. Loss of CDK8 is associated with pronounced transcriptional changes, whereas inhibiting CDK8 kinase activity has minimal effects. Gene set enrichment analysis suggests that the mTOR signaling pathway is deregulated in CDK8-deficient cells and, accordingly, these cells are highly sensitive to mTOR inhibitors. Analysis of large cohorts of human ALL and AML patients reveals a significant correlation between the level of CDK8 and of mTOR pathway members. We have synthesized a small molecule YKL-06-101 that combines mTOR inhibition and degradation of CDK8, and induces cell death in human leukemic cells. We propose that simultaneous CDK8 degradation and mTOR inhibition might represent a potential therapeutic strategy for the treatment of ALL patients.

## Abstract (web summary)

Cyclin-dependent kinases are deregulated in blood cancers. Here, the authors show that CDK8, independent of its kinase activity, regulates mTOR signalling for the maintenance of BCR-ABL1^+^ leukemia, and that the dual inhibition of CDK8 and mTOR signalling induces apoptosis in these cells.

## Introduction

Cyclin-dependent kinases (CDKs) are serine/threonine kinases that are regulated by binding to cyclins^1^. CDKs were initially shown to play important roles in cell cycle control. Over time, their broad and diverse roles in many biological processes were uncovered and CDKs are now considered as important players in transcription, metabolism, neuronal differentiation, hematopoiesis, and stem cell self-renewal [reviewed in ref. ^2^]. CDKs comprise two major sub-groups; the first CDK family including CDK1, 2, 4, and 6 is predominantly involved in cell cycle control. The second group comprising CDKs 7 through 13 are modulators of transcriptional processes^3^. CDK6 unifies functions of both groups^4–8^. This dual function may underlie the great success of three independent CDK4/6 inhibitors (palbo-, ribo-, and abemaciclib), which were recently declared as therapeutic breakthrough by the FDA^9^. Besides CDK4/6, also CDK7 and CDK9 have drawn considerable attention as drug targets. Both CDK7 and CDK9 phosphorylate serine residues in the C-terminal tail of RNA Polymerase II. CDK9 regulates transcription of key genes in hematological cancers such as myeloid cell leukemia-1 (MCL-1), B-cell lymphoma extra-long (BCL-x_L_), or X-linked inhibitor of apoptosis protein (XIAP)^10^. CDK7 is considered to drive super-enhancer-(SE)-associated gene expression in neuroblastoma^11^ and T-cell acute lymphoid leukemia (T-ALL)^12^. First-generation CDK inhibitors such as flavopiridol^13^ and dinaciclib^14^ are active in acute myeloid leukemia (AML)^15^ and chronic myeloid leukemia (CML), but clinical benefit is limited due to adverse effects^16,17^.

CDK8 was initially reported to exert transcriptional repressive functions as part of the mediator complex, a core component of the basal transcription machinery. CDK8 and its paralog CDK19 bind to the mediator complex in a mutually exclusive way but both rely on binding of cyclin C (CCNC) for kinase activity^3^. Single knockout of *Cdk8* and *CCNC* results in embryonic lethality at E2.5-3 due to preimplantation defects^18^, whereas conditional deletion of CDK8 in adult mice is surprisingly well tolerated^19^. Recent studies have shown that CDK8 can exert activating functions as a co-regulator of p53^20^ or hypoxia-induced gene expression^21^. STAT transcription factors are among the best-described targets of CDK8^22,23^. Phosphorylation of STAT1^S727^ enhances transcriptional activity and results in interferon (IFN)-induced gene transcription^24^.

The role of CDK8 appears to be divergent and highly context-dependent. In colon cancer^25,26^, melanoma^27^, prostate^28^, and breast cancer^29^, CDK8 accelerates proliferation and migration. In contrast, it acts as a tumor suppressor in endometrial^30^ and intestinal tumors^19^. In some AML cell lines, inhibition of CDK8 via steroidal alkaloid cortistatin A dramatically alters gene expression and blocks cell proliferation. These changes were due to the relief of CDK8-mediated repression of SE-driven transcription^31^.

The BCR-ABL1 fusion protein drives the development of CML and a subset of ALL cases, which are considered a particular therapeutic challenge. Albeit tyrosine kinase inhibitors (TKIs) for the BCR-ABL1 oncoprotein are available, further therapeutic improvement is required^32^. Resistance mechanisms towards TKIs demand the development of therapeutic strategies^33^. Our findings identify CDK8 as a key mediator of BCR-ABL1-driven leukemia. The role of CDK8 goes beyond its kinase activity, suggesting the development of therapeutic strategies towards its kinase-independent functions.

## Results

### CDK8 is essential for survival of BCR-ABL1^p185+^ leukemic cells

**Fig. 1:**
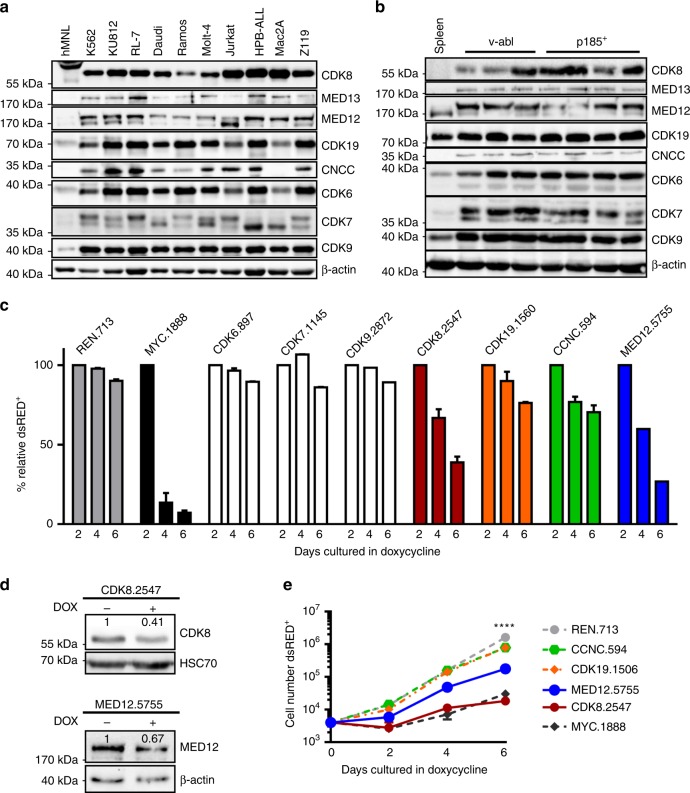
CDK8 is essential for survival of BCR-ABL1^p185+^ leukemic cells. Immunoblotting: levels of CDK6, CDK7, CDK8, CDK9, CDK19, CCNC, MED12, and MED13 in leukemic human (**a**) and murine (**b**) cell lines. Levels of β-actin served as loading control. **c** Induction of shRNA-mediated knockdowns by doxycycline. Percentages of dsRED^+^ BCR-ABL1^p185+^ leukemic cells transduced with TRE3G-dsRED-shRNA-puro (Tet-On) targeting CDK6, CDK7, CDK8, CDK9, CDK19, CNCC, or MED12. Numbers indicate the starting point of shRNA sequence. Data represent frequencies of dsRed^+^ BCR-ABL1^p185+^ cells over time, normalized to the percentages of dsRED^+^ cells after 2 days of doxycycline (DOX) administration. shRNAs directed against Renilla (REN) or MYC served as negative and positive controls. One representative experiment performed in duplicates out of three with similar outcome is shown. **d** Verification of shRNA-mediated knockdown of CDK8 and MED12 by immunoblotting (day 2 after doxycycline administration). β-Actin and HSC70 served as a loading control. Numbers refer to densitometric analysis of the blotted protein in reference to loading control levels. **e** Growth curves of shRNA-expressing (dsRed^+^) Tet-On BCR-ABL1^p185+^ cells. One representative experiment performed in triplicates out of three with similar outcome is shown. Levels of significance were calculated using two-way ANOVA followed by Dunn’s test; data represents means ± SD (****p < 0.0001). Source data are provided as a Source Data file

To investigate which CDKs are expressed in hematopoietic malignancies, we measured the levels of CDK6, CDK7, CDK8, CDK9, and CDK19 in a panel of human leukemic cell lines by immunoblotting. Irrespective of the cells’ origin, the levels of CDK6, CDK7, CDK8, CDK9, and CDK19 were dramatically increased in all cell lines compared with non-transformed human mononuclear lymphocytes (hMNL). CDK8 is part of the kinase submodule of the mediator complex, so we tested whether the other members of this complex are also upregulated and we found increased levels of MED12, MED13, and CCNC, which are part of the mediator kinase module (Fig. 1a). A comparable situation was found in murine leukemia cell lines transformed by the v-ABL^p160+^ or BCR-ABL1^p185+^ oncogenes (Fig. 1b).

We tested which of the CDKs support viability and proliferation of mouse BCR-ABL1^p185+^ B-ALL cells by using an inducible Tet-On RNAi system, in which short hairpin RNA (shRNA) expression is coupled to a dsRed reporter gene. DsRed^+^ cells were detectable 2 days after doxycycline treatment and followed over time. Although knockdown of CDK6, 7, or 9 did not induce strong anti-proliferative responses, we found a pronounced reduction in the frequency of dsRed^+^ cells upon knockdown of CDK8 comparable to MYC knockdown, a positive control (Fig. 1c). This prompted us to include CDK19, which has functional homology to CDK8, and the CDK8-binding partners CCNC and MED12, which regulate CDK8’s kinase activity. Neither of these knockdown approaches fully recapitulated the effects of loss of CDK8, with the exception of MED12 (Fig. 1c). Efficiencies of CDK6, CDK7, CDK9, CDK19, MED12, and CCNC knockdown were verified by immunoblotting (Fig. 1d and Supplementary Fig. 1a). Downregulation of CDK8, MED12, or c-MYC was less pronounced, indicating that loss of either protein is incompatible with survival. Growth curves supported these observations (Fig. 1e).

Stable shRNA-mediated knockdown for CDK9, CDK19, MED12, MED13, or CCNC in four individually derived murine BCR-ABL1^p185+^ cell lines gave similar effects to the inducible knockdowns. However, we failed to obtain lines deficient for CDK8 or CDK7 (Supplementary Fig. 1b). We have no explanation for the different effects of CDK7 deletion in short- and long-term knockdown experiments. Our failure to generate CDK8-deficient lines shows that loss of CDK8 is incompatible with survival of BCR-ABL1^p185+^ cells.

### Steady-state hematopoiesis is not affected by loss of CDK8

**Fig. 2:**
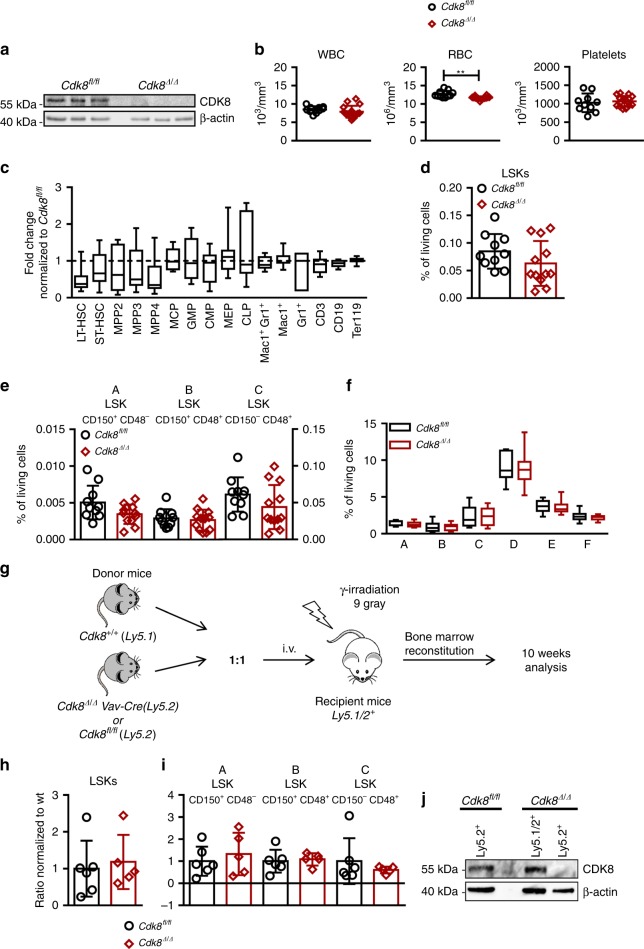
Steady-state hematopoiesis is not affected by loss of CDK8 (*Cdk8*^*Δ/Δ*^*Vav-Cre*). **a** Efficiency of CDK8 deletion in 6-week-old *Cdk8*^*Δ/Δ*^*Vav-Cre* mice. Immunoblotting of BM cells from *Cdk8*^*fl/fl*^ and *Cdk8*^*Δ/Δ*^*Vav-Cre* mice (*n* = 3 per genotype). **b** Analysis of white blood cell count (WBC), red blood cell count (RBC), and platelet count of *Cdk8*^*fl/fl*^ (*n* = 10) and *Cdk8*^*Δ/Δ*^*Vav-Cre* mice (*n* = 16) are depicted. **c** Relative fold change of BM composition of *Cdk8*^*Δ/Δ*^*Vav-Cre* mice (*n* = 10) normalized to mean of *Cdk8*^*fl/fl*^ (*n* = 10). Center value represents median, the box 25th to 75th percentiles, and whiskers min to max. **d** Bar diagram of Lin^−^ Sca-1^+^ c-kit^+^ (LSK) frequencies in BMs of *Cdk8*^*fl/fl*^ (*n* = 10) and *Cdk8*^*Δ/Δ*^*Vav-Cre* (*n* = 12) mice. **e** Frequencies of LSK subpopulations (fraction A, B, and C; *Cdk8*^*fl/fl*^ *n* = 10, *Cdk8*^*Δ/Δ*^*Vav-Cre* *n* = 12). **f** Frequencies of individual populations during early B-cell development according to Hardy nomenclature in pre-pro-B (B220^+^/CD43^hi^/CD19^−^/BP-1^−^; fraction A), early pro-B (B220^+^/CD43^hi^/CD19^+^/BP-1^−^; fraction B), late pro-B (B220^+^/CD43^hi^/CD19^+^/BP-1^+^; fraction C), pre-B (B220^+^/CD43^lo^/IgM^−^/IgD^−^, fraction D), immature (B220^+^/CD43^lo^/IgM^+^/IgD^−^, fraction E), and mature (B220^+^/CD43^lo^/IgM^+^/IgD^+^; fraction F) B cells^34,35^ (*Cdk8*^*fl/fl*^ *n* = 9, *Cdk8*^*Δ/Δ*^Vav-Cre *n* = 12). Center value represents median, the box 25th to 75th percentiles and whiskers min to max. **g** Experimental setup of competition transplants data shown in **h**, **i**. **h** Bar graph displays LSK^+^ cells analyzed for Ly5.1^+^/Ly5.2^+^ composition in total BM. **i** Contributions of Ly5.1^+^ and Ly5.2^+^ cells in LSK subpopulations (fraction A, B, and C; *Cdk8*^*fl/fl*^ *n* = 6 and *Cdk8*^*Δ/Δ*^*Vav-Cre* *n* = 5). **j** Immunoblot of sorted BM cells after competitive transplant. Detection of CDK8 in *Cdk8*^*fl/fl*^ *Ly5.2*, *Cdk8*^*fl/fl*^ *Ly5.1*, and *Cdk8*^*∆/∆*^ *Ly5.2* cells; β-actin served as loading control. Levels of significance were calculated using **b**, **d**, **e**, **f** unpaired *t*-test, **i** Mann–Whitney, and **c** Kruskal–Wallis test followed by Dunn’s test; data represent means ± SD (**p < 0.01). Source data are provided as a Source Data file

Efficient drug treatment requires a sufficiently large therapeutic window, which allows killing of tumor cells while sparing normal tissue. We therefore investigated the consequences of *Cdk8* deletion on normal, non-leukemic hematopoiesis using *Cdk8*^*Δ/Δ*^*Vav-Cre* mice. Bone marrow (BM) was isolated from 6-week-old mice. Efficient deletion of CDK8 was verified by immunoblotting (Fig. 2a). Overall, the loss of CDK8 was well tolerated, as white blood cell counts (WBCs), red blood cell counts (RBCs) and numbers of platelets were comparable to those of control mice (Fig. 2b). Detailed flow cytometric analyses revealed no significant differences in the frequencies of hematopoietic cells at various stages of differentiation, indicating that hematopoiesis remained largely unaffected under steady-state conditions (Fig. 2c). Importantly, percentages of LSK cells and stem cell sub-fractions were comparable (Fig. 2d–e). As BCR-ABL1^p185+^ cells are of B-lymphoid origin, we closely investigated the consequences of CDK8 deficiency on early B-cell development. B-cell developmental stages can be distinguished by differential cell-surface expression of B220, CD43, CD19, BP-1, IgM, and IgD^34,35^. Frequencies of individual B-cell fractions were unaltered in BMs of *Cdk8*^*Δ/Δ*^*Vav-Cre* mice (Fig. 2f). Poly(I:C) treatment in *Cdk8*^*Δ/Δ*^*Mx1Cre* mice was used to challenge stress-induced hematopoiesis as it induces a type I IFN-triggered ubiquitous deletion of *Cdk8*. Poly(I:C) treatment in *Cdk8*^*fl/fl*^*Mx1Cre* mice reflected the observations in *Cdk8*^*Δ/Δ*^*Vav-Cre* mice; we did not detect significant changes in the frequencies of hematopoietic cell subsets in the BM or in the blood cells upon *Cdk8* deletion (Supplementary Fig. 2a-f). To explore the functionality of CDK8-deficient stem cells, we set up a competitive transplant experiment. We mixed BM cells from *Cdk8*^*Δ/Δ*^*Vav-Cre Ly5.2*^*+*^ or *Cdk8*^*fl/fl*^ *Ly5.2*^*+*^ mice with *CDK8*^*+/+*^ *Ly5.1*^*+*^ BM cells in a 1:1 ratio and injected them intravenously (i.v.) into lethally irradiated *Ly5.1/2*^*+*^ mice (Fig. 2g). Ten weeks after transplantation, no differences in the repopulation capacity of stem cells of different genotypes were observed (Fig. 2h, i). The absence of CDK8 was confirmed in sorted BM-derived *Cdk8*^*Δ/Δ*^*Vav-Cre Ly5.2*^*+*^ cells (Fig. 2j). In summary, conditional and inducible loss of CDK8 in the hematopoietic system is well tolerated, underlining the potential of CDK8 as a therapeutic target.

### CDK8 is not required for initial BCR-ABL1^p185+^ transformation

**Fig. 3:**
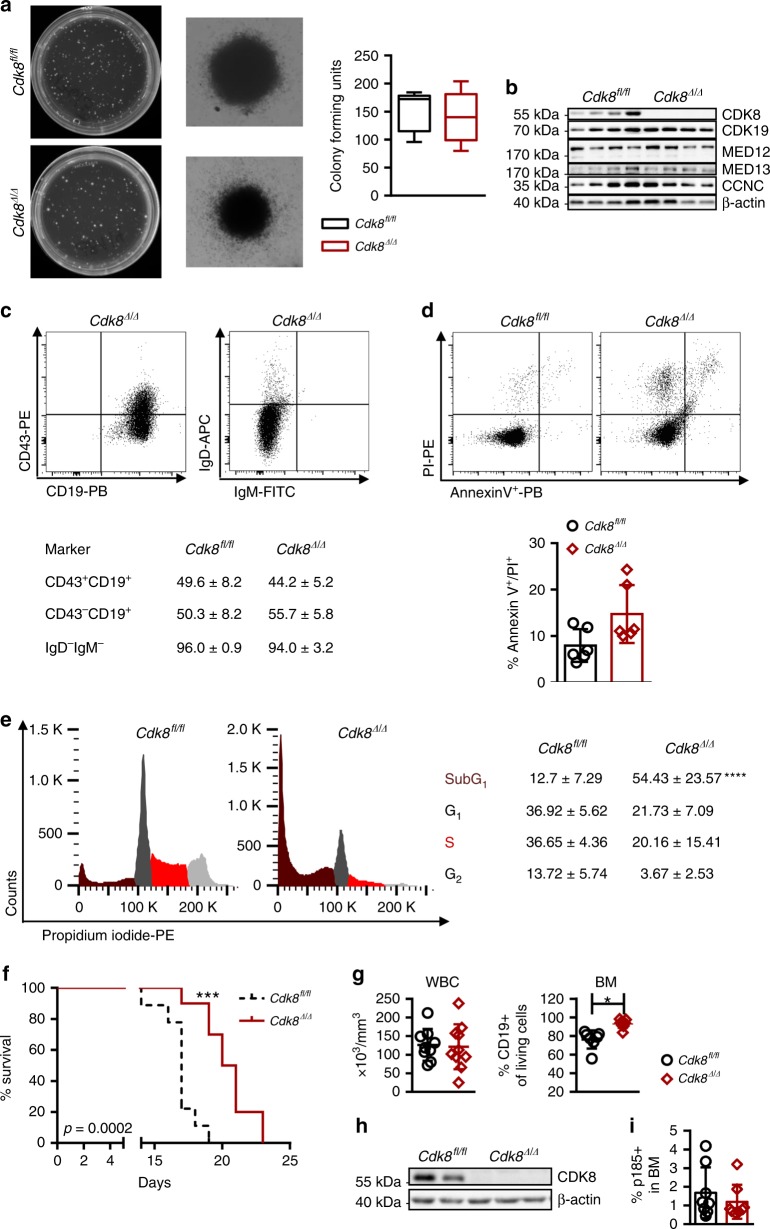
CDK8 is not required for initial BCR-ABL1^p185+^ transformation. **a** BCR-ABL1^p185^-induced colony formation of *Cdk8*^*fl/fl*^ and *Cdk8*^*Δ/Δ*^*Vav-Cre* BM cells in growth-factor-free methylcellulose. The second panel shows single-colony pictures of each phenotype. Summary of colony-formation assays. Center value represents median, the box 25th to 75th percentiles and whiskers min to max (*n* = 4 per genotype; each performed in duplicates). **b** CDK8, CDK19, CCNC, MED12, and MED13 protein levels in BCR-ABL1^p185+^ *Cdk8*^*fl/fl*^ and BCR-ABL1^p185+^ *Cdk8*^*Δ/Δ*^*Vav-Cre* cell lines (immunoblotting). β-Actin served as loading control. **c** Representative FACS blots of B-cell marker staining: B220^+^ gated BCR-ABL1^p185+^ *Cdk8*^*Δ/Δ*^*Vav-Cre* cells with CD43, CD19, IgD, and IgM. Table below indicates frequencies of indicated markers for BCR-ABL1^p185+^ *Cdk8*^*fl/fl*^ and BCR-ABL1^p185+^ *Cdk8*^*Δ/Δ*^*Vav-Cre* cell lines (*n* = 4 per genotype). **d** FACS profile of an AnnexinV/PI staining of BCR-ABL1^p185+^ *Cdk8*^*fl/fl*^ and BCR-ABL1^p185+^ *Cdk8*^*Δ/Δ*^*Vav-Cre* cell lines (*n* = 6 per genotype). **e** PI cell cycle staining of BCR-ABL1^p185+^ *Cdk8*^*fl/fl*^ and BCR-ABL1^p185+^ *Cdk8*^*Δ/Δ*^*Vav-Cre* cell lines. The experiment was performed in technical duplicates; data from one out of three independent experiments are depicted. Table contains frequencies of cells in individual phases of the cell cycle (*n* = 3 per genotype). **f** BCR-ABL1^p185+^ *Cdk8*^*fl/fl*^ and BCR-ABL1^p185+^ *Cdk8*^*Δ/Δ*^*Vav-Cre* cells were injected intravenously (i.v.) into non-irradiated NSG mice (2500 cells/mouse, *n* = 9 mice received BCR-ABL1^p185+^ *Cdk8*^*fl/fl*^ and 10 mice BCR-ABL1^p185+^ *Cdk8*^*Δ/Δ*^*Vav-Cre* cells, 3 independent cell lines per genotype were injected). Survival curves of recipients are depicted (median survival of *Cdk8*^*fl/fl*^ and *Cdk8*^*Δ/Δ*^*Vav-Cre* cohorts: 17 days and 21 days). **g** White blood cell count (WBC) (*n* = 9 *Cdk8*^*fl/fl*^; *n* = 10 *Cdk8*^*Δ/Δ*^*Vav-Cre*) cells and frequencies of CD19^+^ cells in BM of diseased mice (*n* = 7 BCR-ABL1^p185+^ *Cdk8*^*fl/fl*^; *n* = 9 BCR-ABL1^p185+^ *Cdk8*^*Δ/Δ*^*Vav-Cre*). **h** Immunoblotting for CDK8 of ex vivo-derived BCR-ABL1^p185+^ *Cdk8*^*fl/fl*^ and BCR-ABL1^p185+^ *Cdk8*^*Δ/Δ*^*Vav-Cre* cells. Levels of β-actin served as loading control. **i** Homing assay of BCR-ABL1^p185+^ *Cdk8*^*fl/fl*^ vs. BCR-ABL1^p185+^ *Cdk8*^*Δ/Δ*^*Vav-Cre* cells. Bar diagram show percentages of BCR-ABL1^p185+^ cells in the BM 18 h post injection. Asterisks denote statistical significances as determined by an **a**, **c**, **e**, **g** (WBC) unpaired *t*-test, **d**, **g** (BM), **i** Mann–Whitney, or a **f** log-rank test; data represent means ± SD (*p < 0.05; ***p < 0.001; ****p < 0.001). Source data are provided as a Source Data file

To test the consequences of *Cdk8* deficiency for B-lymphoid transformation, we infected *Cdk8*^*Δ/Δ*^*Vav-Cre-*BM cells with a retrovirus encoding pMSCV-*Bcr-Abl1p185*-IRES-eGFP or Ab-MuLV (encoding v-ABL^p160+^). Infected cells were subsequently plated in growth factor-free methylcellulose. Irrespective of the genotype, comparable numbers of B-lymphoid colonies grew out (Fig. 3a and Supplementary Fig. 3a). Deficiency of CDK8 did not affect the levels of CDK19, MED12, MED13, or CNCC (Fig. 3b and Supplementary Fig. 3b). Transformation by BCR-ABL1^p185+^ or v-ABL^p160+^ resulted in the outgrowth of pro-B cells that consistently stain positive for B220, CD19, and CD43, and negative for the maturation markers IgM and IgD (Fig. 3c). BCR-ABL1^p185+^ and v-ABL^p160+^-transduced *Cdk8*^*Δ/Δ*^*Vav-Cre* cell lines showed higher frequencies of apoptotic cells (Fig. 3d and Supplementary Fig. 3c) that were also evident in growth curves; the numbers of v-ABL^p160+^ *Cdk8*^*Δ/Δ*^*Vav–Cre* cells rose significantly slower than those of the control group (Supplementary Fig. 3d). Consistently, the number of cells in the SubG_1_ phase increased upon loss of CDK8 (Fig. 3e and Supplementary Fig. 3e).

The enhanced apoptosis and reduced proliferation of CDK8-deficient cells in vitro may be compensated in vivo by the microenvironment. We transplanted BCR-ABL1^p185+^ or v-ABL^p160+^ transformed *Cdk8*^*Δ/Δ*^ cell lines into NSG mice. Leukemia latency of BCR-ABL1^p185+^ and v-ABL^p160+^ transformed *Cdk8*^*Δ/Δ*^ cells was significantly increased compared to wild-type cells (Fig. 3f and Supplementary Fig. 3f). Analysis of moribund animals unraveled slight differences in disease phenotypes; although the numbers of WBCs were comparable, the frequency of CD19^+^ cells containing the transformed cell population in the BM was significantly increased upon CDK8 loss (BCR-ABL1^p185+^) (Fig. 3g and Supplementary Fig. 3g). The absence of CDK8 was confirmed by immunoblotting of ex vivo*-*isolated BCR-ABL1^p185+^- and v-ABL^p160+^-transduced cells (Fig. 3h and Supplementary Fig. 3h). The prolonged disease latency of the *Cdk8*^*Δ/Δ*^ *Vav-Cre*-transplanted cohort does not result from differences in homing to the BM, as there were no significant differences between BCR-ABL1^p185+^ CDK8-expressing and BCR-ABL1^p185+^ *Cdk8*^*Δ/Δ*^ *Vav-Cre* cells (Fig. 3i). Expression of a CDK8 kinase-dead mutant (D173A) in leukemic *Cdk8*^*Δ/Δ*^ *Vav-Cre* cells accelerated disease development, which indicates that CDK8 exerts its effect in a kinase-independent manner (Supplementary Fig. 3i, j). The data suggest that CDK8 is largely dispensable for the initiation of BCR-ABL1^p185+^ or v-ABL^p160+^ B-ALL, but instead may play an important part in disease maintenance by controlling cell survival.

### CDK8 is required for maintenance of BCR-ABL1^p185+^ leukemia

**Fig. 4:**
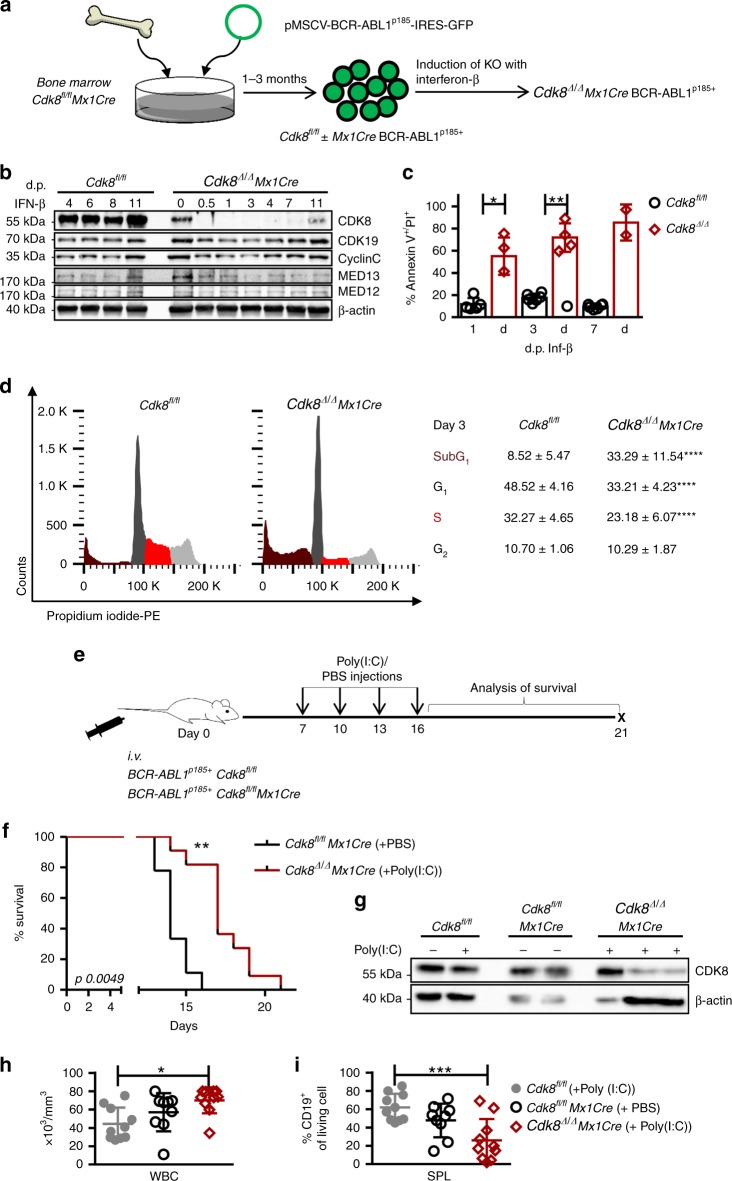
CDK8 is required for maintenance of BCR-ABL1^p185+^ leukemia. **a** Generation of stable BCR-ABL1^p185+^ *Cdk8*^*fl/fl*^ and BCR-ABL1^p185+^ *Cdk8*^*fl/fl*^Mx1Cre cell lines. Scheme depicts experimental setup of data shown in **b**–**d**. **b** Efficiency of CDK8 deletion and protein levels of CDK19, CCNC, MED12, and MED13 at indicated time points post interferon-β (IFN-β) administration (immunoblotting). Levels of β-actin served as a loading control. **c** Proportions of AnnexinV^+^ cells in BCR-ABL1^p185+^ *Cdk8*^*fl/fl*^ or BCR-ABL1^p185+^ *Cdk8*^*Δ/Δ*^ *Mx1Cre* cell lines 1, 3, and 7 days after administration of IFN-β (d.p. IFN-β: days post IFN-β; *n* = 6 BCR-ABL1^p185+^ *Cdk8*^*fl/fl*^; *n* = 4 BCR-ABL1^p185+^ *Cdk8*^*Δ/Δ*^*Mx1Cre*, three independent experiments). **d** Representative PI cell cycle staining of BCR-ABL1^p185+^ *Cdk8*^*fl/fl*^ and BCR-ABL1^p185+^ *Cdk8*^*Δ/Δ*^*Mx1Cre* cell lines. The experiment was performed in duplicates; one of three experiments is depicted. Table indicates frequencies of cells in individual phases of the cell cycle (*n* = 3 per genotype, measured in duplicates). **e** Scheme depicting experimental setup of in vivo experiment. **f** Kaplan–Meier shows survival curves of non-irradiated NSG mice that have received BCR-ABL1^p185+^ *Cdk8*^*fl/fl*^ or BCR-ABL1^p185+^ *Cdk8*^*fl/fl*^*Mx1Cre* cell lines (2500 cells/mouse, *n* = 9 mice received BCR-ABL1^p185+^ *Cdk8*^*fl/fl*^ and *n* = 11 BCR-ABL1^p185+^ *Cdk8*^*fl/fl*^*Mx1Cre* cells, 3 independent cells lines per genotype were injected). **g** Immunoblotting for CDK8 of ex vivo-derived BCR-ABL1^p185+^ cell lines (±poly(I:C) injections). Levels of HSC70 served as a loading control. **h** White blood cell count (WBC) of mice on day of terminal disease (analysis). **i** Summary of frequencies of CD19^+^ cells in spleens (SPL) of diseased mice, *n* = 10 per genotype. Asterisks denote statistical significances as determined by **d** unpaired *t*-test, **c**, **h** Mann–Whitney, **f** log-rank test, or **i** one-way ANOVA followed by Tukey’s test; data represent means ± SD (*p < 0.05; **p < 0.01; ***p < 0.001). Source data are provided as a Source Data file

To investigate the immediate consequence of CDK8 loss in leukemic cells, we created *Cdk8*^*fl/fl*^*Mx1Cre* BCR-ABL1^p185+^ cell lines and deleted *Cdk8* at specific time points by IFN-β (Fig. 4a). Immunoblotting confirmed that IFN-β administration deleted *Cdk8* until day 7, after which multiplication of the few remaining *Cdk8*-positive (non-deleting) cells gave rise to a weak signal for CDK8 (Fig. 4b). Whereas wild-type BCR-ABL1^p185+^ cells were only marginally affected by IFN-β stimulation, the percentage of AnnexinV-positive cells was significantly over 7 days upon loss of CDK8 (Fig. 4c). Cell cycle analysis on day 3 after addition of IFN-β revealed a significant increase of cells in SubG_1_ and a reduction of cells in the S- and G_2_ phases (Fig. 4d). To test the effects of deletion of *Cdk8* in vivo, we transplanted BCR-ABL1^p185+^ wild type and *Cdk8*^*fl/fl*^*Mx1Cre* into NSG mice (Fig. 4e). Deletion of *Cdk8* resulted in a significant survival benefit (Fig. 4f). Immunoblotting of the leukemic cells ex vivo revealed residual CDK8 expression in the leukemic compartment, representing heterozygous deleters or repopulating BCR-ABL1^p185+^ *Cdk8*^*fl/fl*^*Mx1Cre* cells that managed to avoid deletion (Fig. 4g). These non-deleters caused the fatal leukemia with a significant increase in circulating WBCs but a decreased frequency of CD19^+^ cells in the spleen (SPL) (Fig. 4h, i). To follow the kinetics of leukemic cells, we again injected BCR-ABL1^p185+^ *Cdk8*^*fl/fl*^*Mx1Cre* into NSG mice and found a pronounced decrease in the BM upon poly(I:C) treatment after 17 days. Leukemic cells increased again thereafter, indicating a selection of non-deleters that finally induce disease (Supplementary Fig. 4a, b). These experiments using inducible deletion led us to conclude that BCR-ABL1^p185+^ cells depend on CDK8.

### Kinase inhibition fails to mimic the effects of *Cdk8* deletion

**Fig. 5:**
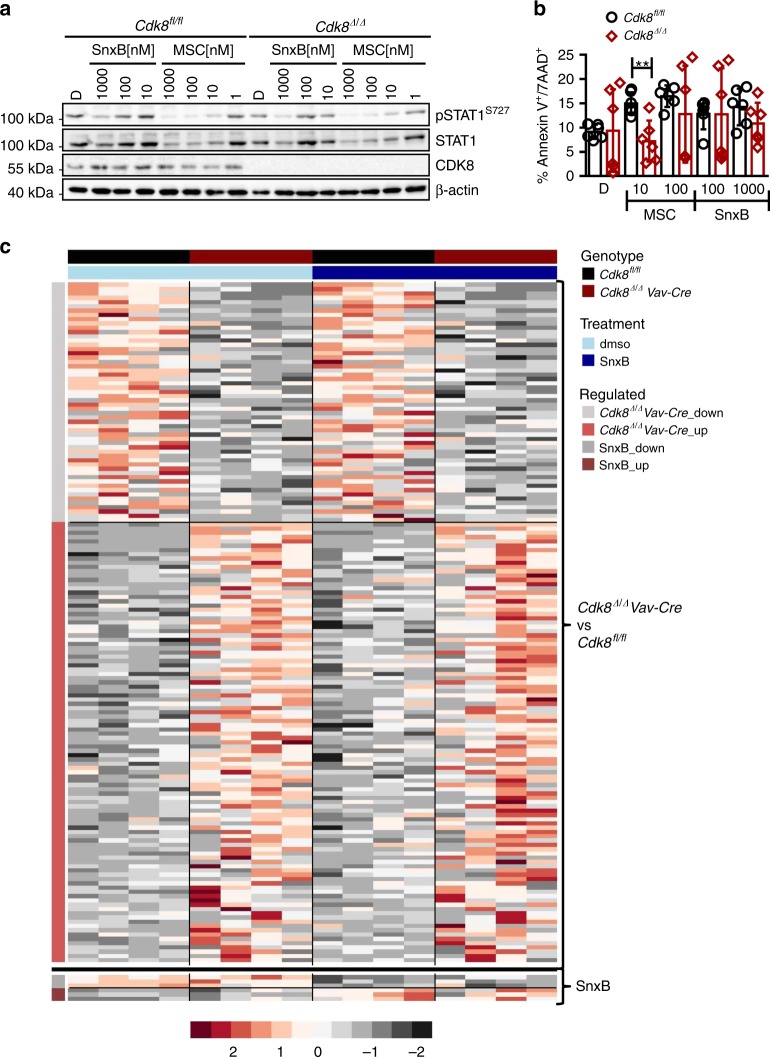
Kinase inhibition fails to mimic the effects of *Cdk8* deletion. **a** Immunoblotting of pSTAT1^S727^ of BCR-ABL1^p185+^ cells after 48 h incubation with increasing concentrations of MSC or Senexin B (SnxB). Induction of phosphorylation was induced by 30 min IFN-β stimulation prior collection. β-Actin served as a loading control. **b** AnnexinV/PI staining (after 48 h) of BCR-ABL1^p185+^ *Cdk8*^*fl/fl*^ and BCR-ABL1^p185+^ *Cdk8*^*Δ/Δ*^*Vav-Cre* cell lines in the presence of indicated Senexin B or MSC concentrations. DMSO (0.1%) served as solvent control. Bars represent means ± SD from two independent experiments (*n* = 2 per genotype, measure in triplicates). Asterisks denote statistical significances as determined by unpaired *t*-test (**p < 0.01). **c** Heatmap of 159 differentially expressed genes between BCR-ABL1^p185+^ *Cdk8*^*fl/fl*^ and BCR-ABL1^p185+^ *Cdk8*^*Δ/Δ*^*Vav-Cre* cell lines (*n* = 4 per genotype). The black line separates the sets of genes, which are differentially regulated upon loss of the entire CDK8 protein (BCR-ABL1^p185+^ *Cdk8*^*Δ/Δ*^*Vav-Cre* cell lines vs. BCR-ABL1^p185+^ *Cdk8*^*fl/fl*^; above the black line) from those whose expressions change upon treatment with 1000 nM Senexin B for 48 h (of BCR-ABL1^p185+^ *Cdk8*^*fl/fl*^ cell lines) (FDR < 0.1). Colors display centered and scaled r-log counts ranging from red to gray (high to low expression). Source data are provided as a Source Data file

Various small-molecule inhibitors have been developed to target CDKs in cancer. To further investigate whether CDK8’s kinase activity accounts for the substantial effects in leukemogenesis, we applied the CDK8/CDK19 inhibitors Senexin B and MSC (MSC2530818). The efficiency of the inhibitors was confirmed by reduction of STAT1^S727^ phosphorylation, a known CDK8 kinase substrate (Fig. 5a)^24^. The reduced phosphorylation was accompanied by a lower induction of STAT1 target genes (*Mx1*, *Stat1*, *Tap1*, *Gbp2*, *Irf1*, and *Ido1)* upon IFN-β stimulation (Supplementary Fig. 5a). Despite reduction of pSTAT1^S727^ and its target genes, there was no significant change in the frequency of apoptotic cells in BCR-ABL1^p185+^ *Cdk8*^*fl/fl*^ and *Cdk8*^*Δ/Δ*^*Vav-Cre* cell lines upon treatment with inhibitor (Fig. 5b). The IC_50_ values for Senexin B were almost identical, irrespective of the presence of CDK8 (Supplementary Fig. 5b). We investigated these differences by RNA sequencing (RNA-seq), comparing four individually derived BCR-ABL1^p185+^ *Cdk8*^*Δ/Δ*^*Vav–Cre* cell lines with four BCR-ABL1^p185+^ *Cdk8*^*fl/fl*^ cell lines in the absence and in the presence of a kinase inhibitor (Senexin B). The absence of CDK8 protein resulted in 103 upregulated and 56 downregulated genes in BCR-ABL1^p185+^ *Cdk8*^*Δ/Δ*^*Vav–Cre* compared with control cell lines. In contrast, Senexin B had only minor effects and affected the transcription of only six genes (Fig. 5c and Supplementary Fig. 5c). CDK8 deletion is thus not recapitulated by inhibition of CDK8 kinase activity in leukemogenesis.

### CDK8 regulates the mTOR pathway

**Fig. 6:**
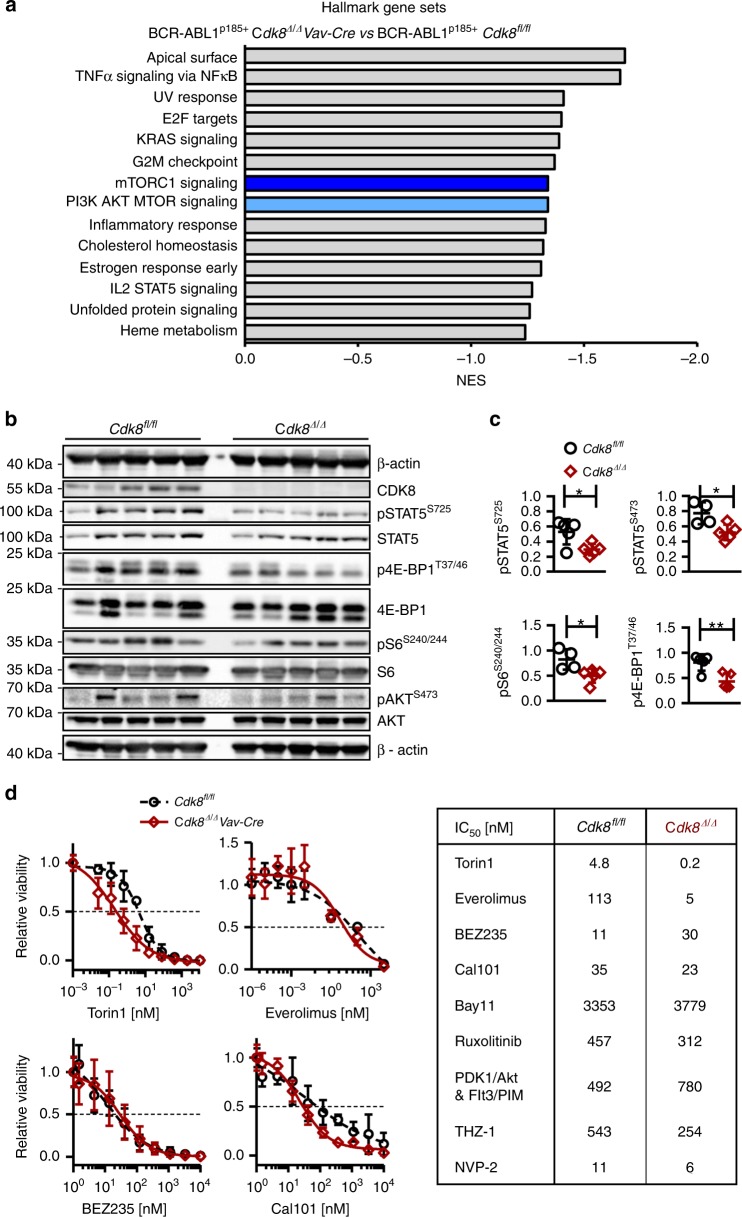
CDK8 regulates the mTOR pathway. **a** Gene set enrichment analysis (RNA-seq data) reveals 14 significantly downregulated pathways in BCR-ABL1^p185+^ *Cdk8*^*Δ/Δ*^*Vav-Cre* cells. NES: normalized enrichment score. **b** Immunoblots of five independent cell lines per genotype were probed for pAKT^S473^, total AKT, pS6^S240/244^, total S6, p4E-BP^T34/46^, and total 4E-BP-1. In addition, pSTAT5^S725^, STAT5, and CDK8 were blotted. β-Actin served as loading control. **c** Bar diagrams depict densitometric analysis of signal intensities relative to the loading control (of western blottings shown in **b)**. **d** Representative dose–response curves for Torin1, Everolimus, BEZ235, and Cal101; mean ± SD. Table includes IC_50_ values of Torin1 (mTORC1 and mTORC2), Everolimus (mTORC1), BEZ235 (PI3K/ATM/ATR and mTOR), Cal101 (PI3Kdelta), Bay11 (NFκB), Ruxolitinib (JAK1 and JAK2), PDK1/Akt and Flt3/Pim dual inhibitor, THZ-1 (CDK7) and NVP-2 (CDK9) on BCR-ABL1^p185+^ *Cdk8*^*fl/fl*^, and BCR-ABL1^p185+^ *Cdk8*^*Δ/Δ*^*Vav-Cre* cell lines. Data represent the summary of one to three cell lines per genotype in technical triplicates. Asterisks denote statistical significances as determined by an unpaired *t*-test; mean ± SD (*p < 0.05; **p < 0.01). Source data are provided as a Source Data file

We used gene set enrichment analysis (GSEA) to identify pathways that are perturbed in the absence of CDK8 (*Cdk8*^*Δ/Δ*^*Vav–Cre*). GSEA analysis identified downregulation of 14 pathways, including several prominent signaling pathways involved in BCR-ABL1-driven disease. Among the top hits, we found “tumor necrosis factor-α signaling via nuclear factor-κB (NFκB)”, “mammalian target of rapamycin complex 1 (mTORC1) signaling,” and “phosphoinositide 3-kinase (PI3K) AKT mTOR signaling” (Fig. 6a). A complete list of deregulated pathways is provided in Supplementary Table 1. Confirmation was provided by the EnrichR program (Supplementary Fig. 6a and Supplementary Table 2), which validated the deregulation of 18 genes (Supplementary Fig. 6b). Phosphorylation of key players in the mTOR pathway (pS6^S240/244^, p4E-BP-1^T37/46^, and pAKT^S473^) was reduced in CDK8-deficient cells (Fig. 6b, c). We confirmed the involvement of CDK8 in the regulation of signaling pathways with commercially available inhibitors of the mTOR, PI3K, and NFκB pathways. IC_50_ levels were determined in BCR-ABL1^p185+^ *Cdk8*^*fl/fl*^ and BCR-ABL1^p185+^ *Cdk8*^*Δ/Δ*^*Vav–Cre* cell lines (Fig. 6d). Loss of CDK8 was associated with a >20-fold enhanced susceptibility to the two mTOR inhibitors Torin1 and Everolimus. The combined inhibition of PI3K and mTOR (by BEZ235) was threefold more effective in *Cdk8*-positive cell lines, presumably because of feedback loops. Sensitivity to inhibition of the NFκB pathway (by Bay11) was little altered by the loss of CDK8 and the effects of inhibition of PDK1/Akt, Flt3/PIM, JAK1, and JAK2 (by Ruxolitinib), and of PI3Kδ (by Cal101) were also independent of the presence of CDK8. The absence of CDK8 renders cells slightly more susceptible to inhibition of CDK9 (NVP-2) or CDK7 (THZ-1), although the IC50’s are well above the published ranges for specific effects so the results presumably reflect general toxicity (Fig. 6d and Supplementary Fig. 6c). The findings confirm that CDK8 has a role in the mTOR signaling pathway.

### Chemical CDK8 degradation cooperates with mTOR inhibition

**Fig. 7:**
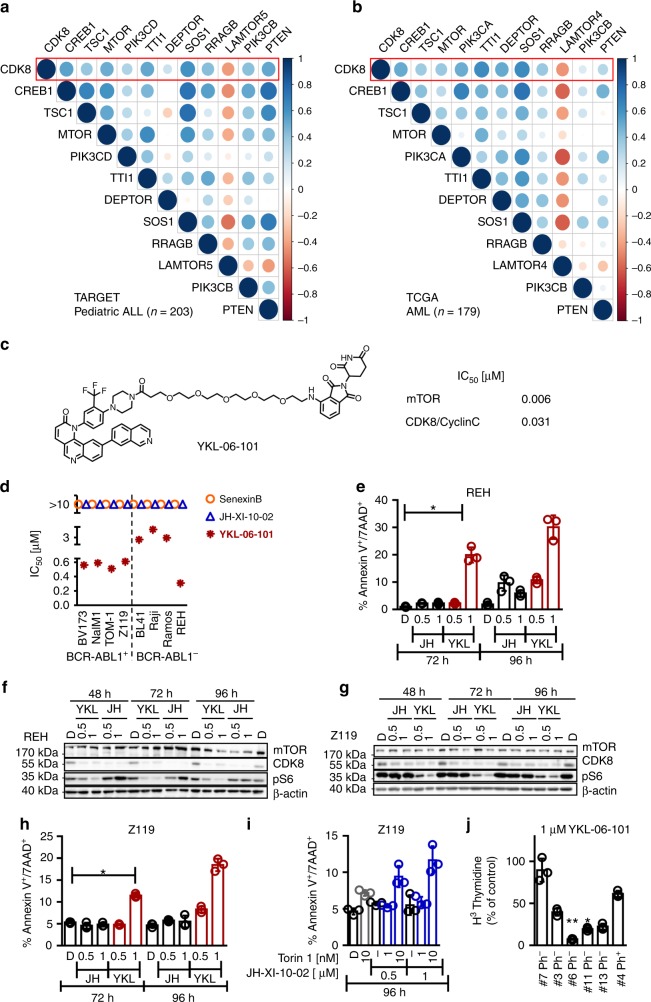
Chemical CDK8 degradation cooperates with mTOR inhibition. **a** Co-expression matrix of CDK8 and members of mTOR/PI3K signaling pathways in samples of 203 pediatric ALL patients (TARGET cohort, RNA-seq) and **b** 179 AML patients (TCGA AML cohort, RNA-seq). The scale indicates Spearman’s correlation coefficients ranging from −1 (red) to 1 (blue). **c** Structure of degrader YKL-06-101 and biochemically verified IC_50_’s for mTOR and CDK8/Cyclin C inhibition. **d** Thymidine incorporation assay based IC_50_’s of BCR-ABL1^+^ (BV173, Nalm1, Tom1, Z119) and BCR-ABL1^−^ (BL41, Raji, Ramos, REH) human leukemic cell lines after 48 h incubation with increasing concentrations of JH-XI-10-02, YKL-06-101, or Senexin B. **e** AnnexinV/7AAD staining of REH cells incubated with 0.5 µM and 1 µM of JH-XI-10-02 or YKL-06-101 for 72 and 96 h. DMSO was used as vehicle control (D: DMSO, *n* = 3). Immunoblots show mTOR, CDK8, and pS6 after incubation of **f** REH cells or **g** Z119 cells with 0.5 µM and 1 µM of JH-XI-10-02 or YKL-06-101 for 48, 72, and 96 h. DMSO (indicated as D) was used as vehicle control and β-actin served as loading control. **h** AnnexinV/7AAD staining of Z119 cells incubated with 0.5 µM and 1 µM of JH-XI-10-02 or YKL-06-101 for 72 and 96 h. DMSO was used as a vehicle control (D: DMSO, *n* = 3). **i** AnnexinV/7AAD staining of Z119 cells incubated with Torin1, JH-XI-10-02, or both in combination with indicated concentrations for 72 and 96 h. DMSO (indicated as D) was used as a vehicle control. One representative experiment out of three is depicted. **j** Bar diagram depicts relative thymidine incorporation of six primary B-ALL patient samples incubated for 48 h with 1 μM of YKL-06-101. Patient #1 represents a non-responder, patient #4 was BCR-ABL1^+^ (Ph^+^), whereas the others were BCR-ABL1^−^ (Ph^−^). Levels of significance were calculated using Kruskal–Wallis test followed by Dunn’s test, data represents means ± SD (*p < 0.05, **p < 0.01). Source data are provided as a Source Data file

We analyzed publicly available data from large cohorts of ALL (*n* = 203; TARGET study) and AML (*n* = 179; The Cancer Genome Atlas (TCGA) study) patients for a potential link between CDK8 and members of the mTOR pathway. Data on AML patients were included, as CDK8 has been implicated in the disease^31^. In both diseases, we found a significant correlation of CDK8 with members of the mTOR pathway including CREB1, TSC1, mTOR, PIK3CD, ZZI1, DEPTOR, SOS1, RRAGB, LAMTOR5, PIK3CB, and PTEN (Fig. 7a, b). Furthermore, CDK8 and members of the mTOR pathway are correlated in a number of other cancers including thyroid carcinoma, thymoma, prostate adenocarcinoma, and liver hepatocellular carcinoma (Supplementary Fig. 7a).

To evaluate the therapeutic potential of our findings, we targeted CDK8 alone or in combination with mTOR inhibition in human leukemic cells (cell lines or primary samples). As inhibition of CDK8 kinase activity does not mimic the effects of CDK8 knockdown in mice, we used chemical-induced protein degradation strategy^36^ to deplete the CDK8 protein in human leukemic cell lines. The CDK8 degrader JH-XI-10-02 selectively degrades CDK8^37^. In addition, medical chemistry optimization of Torin1, an inhibitor of mTOR, which also affects CDK8 kinase activity (IC_50_ of 159 nM)^38^, resulted in the development of THZ4-55, a more potent inhibitor of CDK8 (IC_50_ = 5 nM). By linking THZ4-55 with thalidomide, a ligand that can recruit the E3 ligase cereblon, we generated a bivalent CDK8 degrader, YKL-06-101, which can also inhibit mTOR (Fig. 7c). We tested Senexin B, JH-XI-10-02, and YKL-06-101 in human BCR-ABL1^+^ (BV173, Nalm1, TOM-1, and Z119) and BCR-ABL1^−^ (BL41, Raji, Ramos, and REH) cells. Degrader JH-XI-10-02 and Senexin B treatment induces a proliferation arrest at IC_50_ values above 10 µM. In contrast, the bivalent degrader YKL-06-101 stops proliferation of BCR-ABL1^+^ (Z119, BV173) and of BCR-ABL1^−^ cells (REH) at concentrations below 1 µM (Fig. 7d). Growth arrest is observed after 48 h (Fig. 7d) and is followed by induction of apoptosis and a significant increase in AnnexinV^+^/7AAD^+^ cells (Fig. 7e, h and Supplementary Fig. 8a). Degradation of CDK8 but not of mTOR was verified by western blotting (Fig. 7f, g and Supplementary Fig. 8b). The reduced phosphorylation of S6^S240/244^, indicative of mTOR inhibition, confirms the dual function of the YKL-06-101 degrader (Fig. 7f, g and Supplementary Fig. 8b). Levels of CDK19, CCNC, MED12, and MED13 remained unaltered, irrespective of whether the CDK8 protein was degraded or its kinase activity was inhibited (Supplementary Fig. 8c, d). We verified the synergistic effects of CDK8 degradation and mTOR inhibition by treating cells simultaneously with the CDK8 degrader JH-XI-10-02 and Torin1. Incubation of BCR-ABL1^+^ Z119 cells with 1 µM JH-XI-10-02 and 10 nM Torin1 caused significantly more apoptosis than single treatments, confirming that the effects result from the simultaneous degradation of CDK8 and the inhibition of mTOR (Fig. 7i).

Treatment of primary ALL patient samples with JH-XI-10-02 and YKL-06-101 confirmed the efficacy of YKL-06-101 but not of JH-XI-10-02 alone to induce apoptosis. YKL-06-101 reduced cell viability in 5 out of 12 patient samples, whereas JH-XI-10-02 had no effect (Fig. 7j and Supplementary Table 3). The result strongly suggests that combining CDK8 degradation with mTOR inhibition may represent a therapeutic approach for a subset of ALL patients.

## Discussion

CDK8 has attracted much attention over the last years as potential target for cancer therapy^39^. Small-molecule inhibitors that block CDK8 kinase activity have been shown to be effective in AML^31^, melanoma^27^, breast^29^, prostate^28^, and colon cancer models^39,40^. We describe a kinase-independent function of CDK8 in hematopoietic tumors and suggest that the proteasome-induced degradation of CDK8 in combination with inhibition of mTOR might represent an addition to the therapeutic armory.

CDKs are present at high levels in hematopoietic neoplasms. Although murine leukemia cells (BCR-ABL1^p185+^) contain high levels of CDK6, CDK7, CDK8, CDK9, and CDK19, knockdown of CDK6, CDK7, CDK9, or CDK19 has little effect on cell survival and proliferation in an inducible system. In contrast, deletion of CDK8 has a dramatic effect. Stable knockdown experiments recapitulated the results with inducible shRNAs, although we were not able to generate CDK7- or CDK8-deficient cell lines. The apparent discrepancy between the short- and long-term effects of CDK7 knockdown can be explained by speculating the existence of a stable factor that depends on CDK7; further experiments will be required to test this notion. The pronounced anti-proliferative effects of *Cdk8* deletion in murine leukemia cells were unexpected and indicate the unique role of CDK8 downstream of BCR-ABL1^p185+^ in murine B-ALL. The effect is at least partially independent of the mediator as the deletion of CCNC or MED13 was well tolerated. The pronounced effects of deleting MED12 may be explained by the requirement for MED12 in hematopoietic stem cell homeostasis: *Med12*^*fl/fl*^*Vav1-Cre* animals die within 2 weeks of birth with severely reduced BM and thymus cellularity^41^.

CDK8 does not influence initial transformation by the BCR-ABL1^p185+^ oncogene but is indispensable for the maintenance of established leukemic cell lines. This effect might stem at least in part from CDK8’s role in the phosphorylation of STAT5, a key factor for leukemia maintenance^22,24,42^. Similar to STAT5, CDK8 is essential for the survival of leukemic cells, making it a potential therapeutic target. Deletion of *Cdk8* after disease onset is associated with reduced viability of leukemic cells in vitro and with prolonged survival of the mice in vivo. An additional benefit from targeting CDK8 might arise from the fact that CDK8 represses the natural killer (NK) cell-dependent surveillance of hematopoietic tumors^23^. Deletion of CDK8 in NK cells enhances their cytotoxicity and prolongs the survival of mice suffering from BCR-ABL1^+^ leukemia^43^. Degrading CDK8 would thus kill two birds with one stone: it would inhibit the survival of leukemic cells, while enhancing NK cell cytotoxicity.

A pre-requisite for drug development is the existence of a sufficiently large therapeutic window—any therapeutic strategy must harm transformed cells, while sparing their healthy counterparts. Although CDK8 is absolutely required for the preimplantation of the embryo, *Cdk8* deletion is well tolerated in adult mice. It does not seem to interfere with homeostasis of hematopoietic organs, irrespective of whether it is performed by means of Mx1Cre or by Vav-Cre, and has no impact on the viability or functionality of the HSC. Our findings are consistent with previous work, in which deletion of *Cdk8* in adult mice had no significant effect^19^. It is possible that CDK19, the paralog of CDK8, can compensate for the loss of CDK8 in the adult organism in non-transformed tissues, as proposed in NK^43^ and prostate cancer cells^28^.

Despite its involvement in the transcription machinery (it associates with the mediator complex), CDK8 appears to have some functions that are context-specific. In AML cell lines, inhibition of CDK8/CDK19 kinase activity upregulates SE-associated genes with tumor suppressor and lineage-controlling functions, thereby exerting anti-leukemic effects^31^. No such effects were seen in HCT116 colon cancer cells^31^ and we do not find them in B-ALL cells. Further studies are required to identify the precise mechanism by which CDK8 influences leukemogenesis. As knockdown of MED13 and CCNC is well tolerated, at least some of the effects are independent of the mediator complex.

Although CDK8 was initially discovered as a kinase that regulates transcription, we provide evidence that it has a function that is independent of its kinase activity: inhibition of the CDK8 kinase activity does not consistently mirror the effects of *Cdk8* gene knockdown. Kinase inhibition with Senexin B or MSC causes only minor effects on transcription, whereas the deletion of CDK8 significantly affects transcriptional responses and critical signaling pathways in BCR-ABL1^p185+^-driven leukemia. This finding is consistent with work in HCT116 colon cancer cells, in which inhibition of CDK8 kinase activity induces weak anti-proliferative responses, whereas gene deletion has pronounced effects^5,25,31,44^. Deletion of *Cdk8* (but not inhibition of its kinase activity) leads to reduced levels of mTOR signaling, which has been implicated in BCR-ABL1^+^ leukemia^45^. Although loss of CDK8 and mTOR inhibition has synergistic effects, not such interdependence is seen between the loss of CDK8 and PI3K inhibition. This apparently paradoxical result presumably stems from the complex feedback loops and crosstalk between the mTOR and other PI3K-dependent pathways. CDK8 has been suggested to interact with mTOR in the context of lipogenesis^46^ but not in any form of cancer. An analysis of publicly available RNA-seq data from a range of human cancers suggests that CDK8 levels are highly correlated with the levels of members of the mTOR pathway. The mechanism for the interaction remains a matter for conjecture: we have no direct evidence for or against a physical interaction between CDK8 and mTOR.

TKIs have revolutionized leukemia therapy. Nevertheless, the prognosis for many types of cancer remains poor and patients face a high risk of acquiring resistance-mediating mutations. Specific protein degradation represents a recent mechanism to target proteins independent of their enzymatic activity. We have investigated the potential application of CDK8 degraders, testing the effects of two structurally distinct compounds in human leukemic cell lines. Both molecules cause the efficient degradation of CDK8 but only YKL-06-101 is able to block mTOR signaling. The combined effect of degrading CDK8 and inhibiting mTOR significantly increased apoptosis in three human leukemic cell lines. We believe that this strategy holds a great promise for the clinic, as targeting two unrelated signaling pathways will reduce the probability of developing resistance. Our preliminary investigations suggest that CDK8 interacts with the mTOR signaling pathway not only in a subset of leukemia patients but also in a range of solid cancers. We therefore propose that a combinatorial therapy could represent an approach to treat a range of human cancers.

## Methods

### Cell culture

The following commercially available cell lines were obtained from American Type Culture Collection (ATCC). Established human B-lymphoid cell lines: RL-7 (ATCC no. CRL-2261), REH (ATCC no. CRL-8286), Daudi (ATCC no. CCL-213), and Ramos (ATCC no. CRL-1596). Transformed human T cell lines: Molt-4 (ATCC no. CRL-1582), Jurkat (ATCC no. TIB-152), Mac2A^47^ (RRID: CVCL_H637), and HPB-ALL^48^. K562 (ATCC no. CCL-243) is a well-defined human erythroid leukemia cell line transformed by the BCR-ABL1 oncogene. The following cell lines were kindly provided by Peter Valent and purchased from the Leibnitz Institute DSMZ-German Collection of Microorganisms and Cell Cultures; Ph^+^ cell lines: KU812 (RRID: CVCL_0379), TOM-1 (RRID: CVCL_1895), NALM1 (RRID: CVCL_0091), BV173 (RRID: CVCL_0181); Ph^−^ cell lines: BL41 (RRID: CVCL_1087) and Raji (RRID: CVCL_0511) are Ph^−^. The Ph^+^ Z119 (RRID: CVCL_IU88) cell line was kindly provided to J. V. Melo by Zeev Estrov. hMNLs were isolated from peripheral blood samples over a Ficoll gradient. Human leukemia cell lines and different v-ABL^p160+^, BCR-ABL1^p185+^ mouse cell lines were cultured in RPMI-1640 (Sigma) supplemented with heat-inactivated fetal calf serum (FCS), 50 μM 2-mercaptoethanol, and 100 U/mL penicillin/streptomycin (Sigma). A010 cells (Ab-MuLV producer) and phoenix-ECO (ATCC^®^ CRL-3214^TM^) packaging cells were cultured in Dulbecco’s modified Eagle’s medium (DMEM) (Sigma) medium containing 10% FCS and 100 U/mL penicillin/streptomycin. Cell lines are maintained at 37 °C (5% CO_2_) and have been tested regularly for the absence of *Mycoplasma*.

### shRNA knockdowns

For the inducible knockdown experiments, 20 bp shRNAs against mouse CDK8 (*Cdk8* mRNA (NM_153599); starting position: 2547), CDK6 (*Cdk6* mRNA (NM_009873) starting position: 897), CDK7 (*Cdk7* mRNA (NM_009874) starting position: 1145), CDK9 (*Cdk9* mRNA (NM_130860) starting position: 2872), CDK19 (Cdk19 mRNA (NM_198164) starting position 1560), CCNC (*Ccnc* mRNA (NM_016746) starting position: 594), MED12 (*Med12* mRNA (NM_021521) starting position: 5755), and MED13 (Med13 mRNA (NM_001080931) starting position: 9367) were designed based on improved design rules and cloned into microRNA stem (miR-E) in the pSIN-TRE3G-dsRED-miR-E-PGK-Neo (RT3GEN)-inducible retroviral vector^49^. Two vectors containing shRNA against *Renilla* (Ren.713) and MYC (*Myc* mRNA (NM_001177352) starting position 1888) served as controls. Exact shRNA sequences are available in the Supplementary Table 4.

BCR-ABL1^p185+^ cell lines, modified to express rtTA3^50^, were cultured in RPMI-1640 as above with additional 2 µg/mL Puromycin (Invivogen/Eubio). Tet-On BCR-ABL1^p185+^ cells were transduced with RT3GEN-based retroviral pseudoparticles supplemented with 7 µg/mL polybrene (Sigma). Transduced cells were selected for 2 weeks in 1 mg/mL G418 (Invitrogen). Positively selected cells were treated with 0.5 µg/mL doxycycline (Sigma) to induce expression of the dsRed-miR-E cassette. To monitor proliferation and dsRED expression, induced BCR-ABL1^p185+^ cell lines were seeded in 48-well plates at a concentration of 4000 cells/well/mL. The cells were incubated at 37 °C, 5% CO_2_, were counted every 48 h, and were transferred to a plate with fresh media. The percentage of dsRED^+^ cells was determined by flow cytometry (FACSCanto II BD Biosystems).

### Mouse strains

Conditional C57Bl/6N-*Cdk8*^*fl/fl*^ (*Cdk8*^*tm1c(EUCOMM)Hmgu*^)^43^ were breed to B6N-Tg(*Mx1Cre*)^51^ and B6N-Tg(*Vav-Cre*)^52^. *Cdk8*^*fl/fl*^, *Cdk8*^*fl/fl*^*Mx1Cre*, *Cdk8*^*fl/fl*^*Vav-Cre*, *Ly5.1*^*+*^*(CD45.1*^*+*^*)*, *Ly5.1/2*^*+*^ (CD45.1^+^ and CD45.2^+^), and NSG (NOD.Cg-Prkdc^scid^Il2rg^tm1Wjl^/SzJ; The Jackson Laboratory) were maintained under pathogen-free conditions at the University of Veterinary Medicine Vienna. Genotyping primers and detailed thermocycling conditions are available in the Supplementary Table 5.

### Generation of leukemic cell lines and in vitro deletion of endogenous Cdk8

To generate stable leukemic cell lines, bones of *Cdk8*^*fl/fl*^*Mx1Cre* or *Cdk8*^*fl/fl*^*Vav-Cre* were flushed to isolate BM cells for transformation. The phoenix ecotropic (φNX Eco) packaging system was used to produce supernatant containing pMSCV-IRES-GFP-based BCR-ABL1^p185+^ retroviral pseudoparticles. Therefore, phoenix cells were transfected with retroviral vectors using TurboFect® Transfection Reagent (Qiagen). Cells were seeded 1 day before the transfection in six-well plates and grown to 50–70% confluence. Plasmid DNA (1.5 µg) was diluted in 100 µL DMEM containing 10 mM HEPES buffer pH 7.4. 10 µL Turbofect® Transfection Reagent was added, vortexed, and incubated 15 min at room temperature before the transfection mix was added dropwise to the cells. After 24 h, the transfection mix was replaced by RPMI-1640 supplemented with 10% FCS, 50 μM 2-mercaptoethanol, and 100 U/mL penicillin, 100 μg/mL streptomycin (Sigma). Another 24 h and 48 h later freshly collected BM cells were infected with the collected supernatant. A010 cells were used for the production of an ecotropic replication-deficient form of the Abelson virus. To delete *Cdk8* in the BCR-ABL1^p185+^ *Cdk8*^*fl/fl*^*Mx1Cre* lines, leukemic cells were incubated 24 h in 1000 U/mL recombinant IFN-β (MerckMillipore). *Cdk8* deletion was verified by immunoblot analysis.

### Colony-formation assay

BM cells from 6-week-old donor mice were transduced with Abelson virus or BCR-ABL1^p185+^ supernatants including 7 µg/mL polybrene (Sigma). After 24 h, cells were collected and equal numbers of cells were embedded into growth factor-free methylcellulose (MethoCult™). Colonies were counted after 10–14 days.

### In vivo leukemia studies

A total of 2500 *Cdk8*^*fl/fl*^ BCR-ABL1^p185+^, *Cdk8*^*Δ/Δ*^*Vav-Cre* BCR-ABL1^p185+^, or 1 × 10^5^ *Cdk8*^*fl/fl*^ v-ABL^p160+^ or *Cdk8*^*Δ/Δ*^*Vav-Cre* v-ABL^p160+^ cells were injected via the tail vein into non-irradiated NSG mice. Disease onset was traced by analyzing peripheral blood. Upon appearance of BCR-ABL1^p185+^ cells in the peripheral blood, we initiated poly(I:C) injections (200 µg) to induce Cre recombinase expression and *Cdk8* deletion, specifically in the transplanted leukemic cells. Poly(I:C) injections were given intraperitoneally every 3 days (four times in total); subsequently, survival of the mice was monitored. BM, SPL, and blood were collected, cells isolated in phosphate-buffered saline (PBS), and further analyzed by fluorescence-activated cell sorting (FACS).

### Homing assay

*Cdk8*^*Δ/Δ*^*Vav-Cre Ly5.2*^*+*^ or *Cdk8*^*fl/fl*^ *Ly5.2*^*+*^ BM was mixed with *CDK8*^*+/+*^ *Ly5.1*^*+*^ BM cells in a 1:1 ratio (containing comparable numbers of LSKs) and injected them i.v. into lethally irradiated (9 Gy) *Ly5.1/2*^*+*^ mice. Ten weeks after transplantation, composition of BM of recipient mice was analysed for repopulation of LSKs and stem cell populations by FACS staining.

For the noncompetitive setting, 1 × 10^6^ BCR-ABL1^p185+^ *Cdk8*^*fl/fl*^ *Ly5.2*^+^ or BCR-ABL1^p185+^ *Cdk8*^*Δ/Δ*^*Vav-Cre Ly5.2*^*+*^ cells were injected into lethally irradiated (9 Gy) *Cdk8*^*+/+*^ *Ly5.1*^*+*^ mice. After 18 h, mice were euthanized and the BM was analysed for the presence of BCR-ABL1^p185+^ Ly5.2^+^ cells by FACS.

### Flow cytometry

Single-cell suspensions of the splenocytes, thymus, and BM were prepared. For blood analysis, the erythrocytes were lysed using BD FACS Lysing Solution according to the manufacturer’s protocol (BD Bioscience). Hematopoietic stem cell fractions in the BM were identified according to Wilson et al.^53^. Hematopoietic progenitors were characterized by flow cytometry as follows: MCP (Lin^−^, CD127^−^, c-Kit^+^, Sca-1^−^), CMP (Lin^−^, CD127^−^, CD16/CD32^−^, CD34^+^), GMP (Lin^−^, CD127^−^, CD16/CD32^+^, CD34^+^), MEP (Lin^−^, CD127^−^, CD16/CD32^−^, CD34^−^), and CLP (Lin^−^, CD127^+^, c-kit^mid^, Sca-1^mid^). For cell cycle analysis, cells were stained with propidium iodide (PI) (50 μg/mL) in a hypotonic lysis buffer (0.1% sodium citrate, 0.1% Triton X-100, 100 μg/mL RNAse) and incubated at 37 °C for 30 min. Analysis of apoptotic fractions was performed by staining with AnnexinV and 7AAD or PI in AnnexinV 10× Staining Buffer (eBiosciences) according to the manufacturer’s protocol. Flow cytometry experiments were performed on a BD FACSCanto II (BD Bioscience) and analyzed using BD FACSDiva V8.0 or FlowJo V10 software. The detailed information to utilized antibodies are available in the Supplementary Table 6.

### RNA-seq analysis and GSEA

RNA was isolated from immortalized BCR-ABL1^p185+^ *Cdk8*^*fl/fl*^ and *Cdk8*^*Δ/Δ*^*Vav-Cre* cells. Single-end, 50 bp sequencing of libraries prepared with the Lexogen SENSE mRNA-Seq library preparation kit was performed on an Illumina HiSeq-2500 sequencer. After quality control of raw data with FastQC and removement of adapters and low quality reads with Trimmomatic (version 0.36), reads were mapped to the GENECODE M13 genome using STAR (version 2.5.2b) with default parameters. Then, featureCounts from the Subread package (version 1.5.1) was used to obtain gene counts for union gene models. Differentially expressed (*p*-adjust < 0.05 and fold change > 2) treatment dependent (*Cdk8*^*fl/fl*^ dimethyl sulfoxide (DMSO) vs. *Cdk8*^*fl/fl*^ Senexin B; *Cdk8*^*Δ/Δ*^*Vav-Cre* DMSO vs. *Cdk8*^*Δ/Δ*^*Vav-Cre* Senexin B) and genotype-dependent (*Cdk8*^*fl/fl*^ vs. *Cdk8*^*Δ/Δ*^*Vav Cre*) genes were identified using DESeq2 (version 1.18.1). For the heatmap, centered and scaled regularized log-transformed^54^ library size-normalized counts were visualized using the aheatmap function of the R package NMF (version 0.20.6). The command-line version of GSEA was used for GSEA^55^ and pathway analysis was performed with EnrichR^56^ using either significant up- or downregulated genes (fold change > 2, padjust < 0.1) of *Cdk8*^*fl/fl*^ vs. *Cdk8*^*Δ/Δ*^*Vav Cre* cell lines.

### Co-expression matrix

Publicly available datasets of the TARGET pediatric ALL cohort and of 19 TCGA cohorts were obtained from the cBioPortal for Cancer Genomics database^57,58^. RNA-seq RPKM values of CDK8 and various members of the mTOR/PI3K signaling pathways were extracted from these datasets and were used for co-expression analyses. Spearman’s correlation was calculated using the cor function of R software (v3.5.1) and the resulting correlation matrix was visualized using the corrplot package.

### Immunoblotting

Whole-cell extracts were lysed in RIPA buffer (50 mM Tris-HCL (pH 7.6), 150 mM NaCl, 1% NP-40, 0.25% sodium deoxycholate, 1 mM EDTA; 20 mM β-glycero-phosphate, 1 mM sodium vanadate, 1 mM sodium fluoride, 1 mg/mL aprotinin, 1 mg/mL leupeptin, and 1 mM phenylmethylsulfonyl fluoride) or in SDS-sample buffer. Equal amounts of proteins were separated by SDS polyacrylamide gels and were transferred to nitrocellulose membranes (Whatman^®^Protran^®^). After blocking with 5% bovine serum albumin in pY-TBST buffer (10 mM Tris/HCl pH 7.4, 75 mM NaCl, 1 mM EDTA, 0.1% Tween-20), membranes were probed with primary antibodies (Supplementary Table 7) overnight at 4 °C. Immunoreactive bands were visualized after incubation with the secondary antibody and Clarity Western ECL Substrate (Bio-Rad) using the ChemiDoc^TM^ Touch (Bio-Rad).

### RNA isolation and qRT-PCR

Total RNA was isolated from stable BCR-ABL1^p185+^ cell lines. RNA was extracted using the RNeasy Mini Kit (Qiagen). Reverse transcription was performed using the iSCRIPT cDNA synthesis kit following the manufacturer’s instructions (Bio-Rad). All quantitative PCRs (qPCRs) were performed in triplicates with Sso Advanced Universal SYBR Green Supermix (Bio-Rad) according to the instructions of the manufacturer. Levels of mRNAs were normalized to hypoxanthine guanine phosphoribosyltransferase (HRPT), β-actin, or Ube2d2a mRNA. Primer information are available in the Supplementary Table 8.

### Inhibitors

For dose–response curves, BCR-ABL1^p185+^ *Cdk8*^*fl/fl*^ or *Cdk8*^*Δ/Δ*^*Vav-Cre* cells were plated in triplicates in 96-well plates. Four thousand cells per well in 100 µL of media were incubated 48 h with inhibitors (Senexin B: Gentaur GmBH/APExBio; MSC2530818, THZ-1, Torin1, and Everolimus: Selleck Chemicals; Cal101, PDK1/Akt/Flt dual pathway inhibitor, and BAY11-7085: Calbiochem; NVP-2, SEL120-34A: MedChemExpress; Ruxolitinib: Chemietek). Cell viability was assessed using CellTiterGlo (Promega) according to the manufacturer’s instructions. Data for each cell line were normalized to the negative control (DMSO, set to 100% viability) and 50% inhibitor concentration (IC_50_) was determined by using GraphPad Prism® version 5.00. REH, Z119, BV173, BCR-ABL1^p185+^ *Cdk8*^*fl/fl*^, or *Cdk8*^*Δ/Δ*^*Vav-Cre* cells were seeded in a six-well dish at a concentration of 10^6^ cells/mL. Senexin B, MSC2530818, or SEL120-34A was added and after 48 h incubation cells were collected. Cells (10^6^) were washed with ice-cold phosphate-buffered saline (PBS) and were boiled (95 °C) for 20 min in 100 µL SDS-sample buffer consisting of 5% SDS (Biomol), 5% glycerol (Merck), 2.5% 2-mercapoethanol, and a trace amount of bromophenol blue sodium salt (Merck) in 375 mM Tris/HCl (pH 6.8). The remaining cells were analyzed by FACS (cell cycle and viability).

### Degraders

Synthesis details of YKL-06-101 and JH-XI-10-02 are provided in Supplementary Notes 1 and 2. Proliferation of degrader-exposed human leukemic cell lines and primary ALL patient cells were examined by measuring 3H-thymidine uptake after 48 h. For immunoblottings and FACS analysis, cells were incubated with the indicated concentrations of the degraders up to 96 h.

### Statistical analysis

Kruskal–Wallis test (followed by Dunn’s test), one-way analysis of variance (followed by Tukey’s multiple comparison test), log-rank (Mantel–Cox) test, Wilcoxon–Mann–Whitney test, and assessment of half maximal inhibitory concentration values were performed using GraphPad Prism^®^ Software version 5.04 and 6.02. All data are shown as mean ± SD or otherwise as described in the figure legends. The significance is indicated for each experiment (*p *<* 0.05, **p *<* 0.01, ***p *<* 0.001, ****p < 0.0001).

### Study approvals

All animal experiments were approved by the institutional ethics committee and were granted by the national authority (Austrian Federal Ministry of Science and Research) according to Section 8ff of Law for Animal Experiments under license BMWF-68.205/0218-II/3b/2012, and were conducted according to the guidelines of FELASA and ARRIVE.

The ethics committee of the Medical University of Vienna approved the study on human leukemic cells (approval number: 011-2005). All patients gave their written informed consent to participate before BM or blood cells were examined.

### Reporting summary

Further information on research design is available in the Nature Research Reporting Summary linked to this article.

## Supplementary information

Supplementary Information

Reporting Summary

Source Data

## Data Availability

The RNA sequencing data have been deposited in the Gene Expression Omnibus (GEO) database under the accession code GSE136923. The source data and uncropped gel pictures underlying Figs. 1a–e, 2a–f, h–j, 3a–i, 4b–d, f–i, 5a–b, 6b–d, and 7d–j, and Supplementary Figs. 1a, 2a–f, 3a–h, 4a–b, 5a–b, 6b–c, and 8a–d are provided as a source data file. Data that support the findings of this study are available from the authors upon reasonable request.
