# Supplementary material for: A kinase-independent role for CDK8 in BCR-ABL1+ leukemia
Source: Nat Commun. 2019 Oct 18;10:4741. doi: 10.1038/s41467-019-12656-x (PMC6802219; doi:10.1038/s41467-019-12656-x)
Supplement: Supplementary file 1 — Supplementary Information [file 41467_2019_12656_MOESM1_ESM.pdf]

A kinase-independent role for CDK8 in BCR-ABL1$^+$ leukemia

Menzl et al.

Supplementary Figures

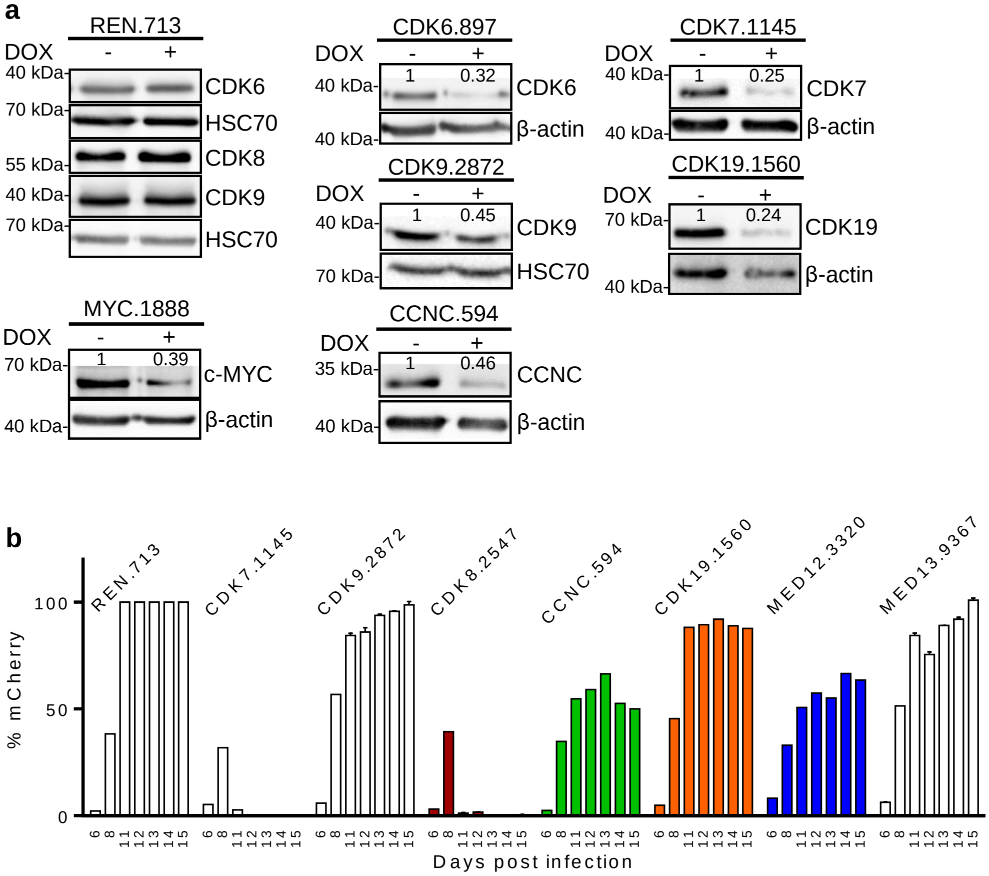

Supplementary Figure 1: CDK8 is essential for survival of BCR-ABL1^{p185+} leukemic cells. **a** Verification of shRNA mediated knockdown in Tet-On BCR-ABL1^{p185+} cell lines. ShREN-expression served as negative and shRNA against MYC served as positive control. Data derived from one representative set of hairpins (directed against REN, MYC, CDK6, CDK7, CDK9, CCNC or CDK19) are depicted. HSC70 or β-actin served as loading controls. **b** Bar diagram shows percentages of mCherry^{+} BCR-ABL1^{p185+} leukemic cells expressing stable shRNAs targeting CDK7, CDK9, CDK8, CCNC, CDK19, MED12 and MED13. Data represents outgrowth of mCherry^{+} BCR-ABL1^{p185+} cells over time. ShRNA directed against Renilla (REN) served as negative control, bars represent mean ± SD. Source data are provided as a Source Data file

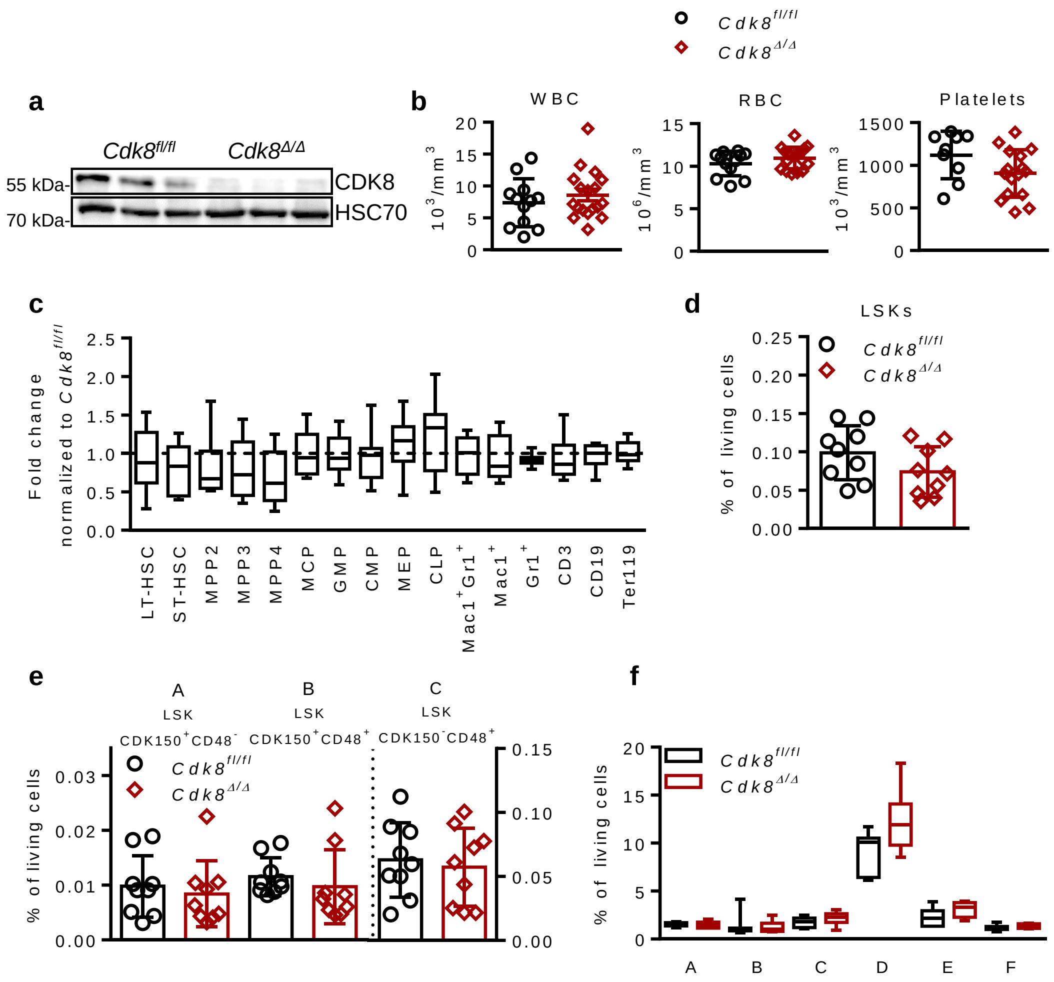

Supplementary Figure 2: Steady-state hematopoiesis is not affected by loss of CDK8 ($Cdk8^{\Delta/\Delta}Mx1Cre$). **a** Efficiency of *Cdk8* deletion in $Cdk8^{\Delta/\Delta}Mx1Cre$ mice after four intraperitoneal poly(I:C) injections. Immunoblot of BM cells from $Cdk8^{fl/fl}$ and $Cdk8^{\Delta/\Delta}Mx1Cre$ mice (n = 3 per genotype). **b** Analysis of white blood cell count (WBC), red blood cell count (RBC) ($Cdk8^{fl/fl}$ n = 12 and $Cdk8^{\Delta/\Delta}Mx1Cre$ mice n = 18) and platelets count of $Cdk8^{fl/fl}$ (n = 9) and $Cdk8^{\Delta/\Delta}Mx1Cre$ mice (n = 16). **c** Relative fold change of BM composition normalized to mean of $Cdk8^{fl/fl}$ BM population frequencies ($Cdk8^{fl/fl}$ n = 11, $Cdk8^{\Delta/\Delta}Mx1Cre$ n = 10). Center value represents median, the box 25th to 75th percentiles and whiskers min to max. **d** Bar diagram of Lin$^-$ Sca-1$^+$ c-kit$^+$ (LSK) frequencies in BM of $Cdk8^{fl/fl}$ (n = 9) and $Cdk8^{\Delta/\Delta}Mx1Cre$ (n = 9) mice. **e** Frequencies of LSK subpopulations (fraction A, B and C; n = 9 per genotype). **f** Frequencies of individual populations during early B-cell development according to Hardy nomenclature in pre-pro-B (B220$^+$/CD43^{hi}/CD19$^-$/BP-1$^-$; fraction A), early pro-B (B220$^+$/CD43^{hi}/CD19$^+$/BP-1$^-$; fraction B), late pro-B (B220$^+$/CD43^{hi}/CD19$^+$/BP-1$^+$; fraction C), pre-B (B220$^+$/CD43^{lo}/IgM$^-$/IgD$^-$, fraction D), immature (B220$^+$/CD43^{lo}/IgM$^+$/IgD$^-$, fraction E) and mature (B220$^+$/CD43^{lo}/IgM$^+$/IgD$^+$; fraction F) B cells ($Cdk8^{fl/fl}$ n = 7 and $Cdk8^{\Delta/\Delta}Mx1Cre$ mice n = 6)[1,2]. Center value represents median, the box 25th to 75th percentiles and whiskers min to max. Levels of significance were calculated using **b** (WBC), **e** (A and B) Mann-Whitney, **c**, **f** Kruskal-Wallis test followed by Dunn's test and **b** (RBC, Platelets), **d**, **e** (C) unpaired *t*-test, data represents means ± SD. Source data are provided as a Source Data file

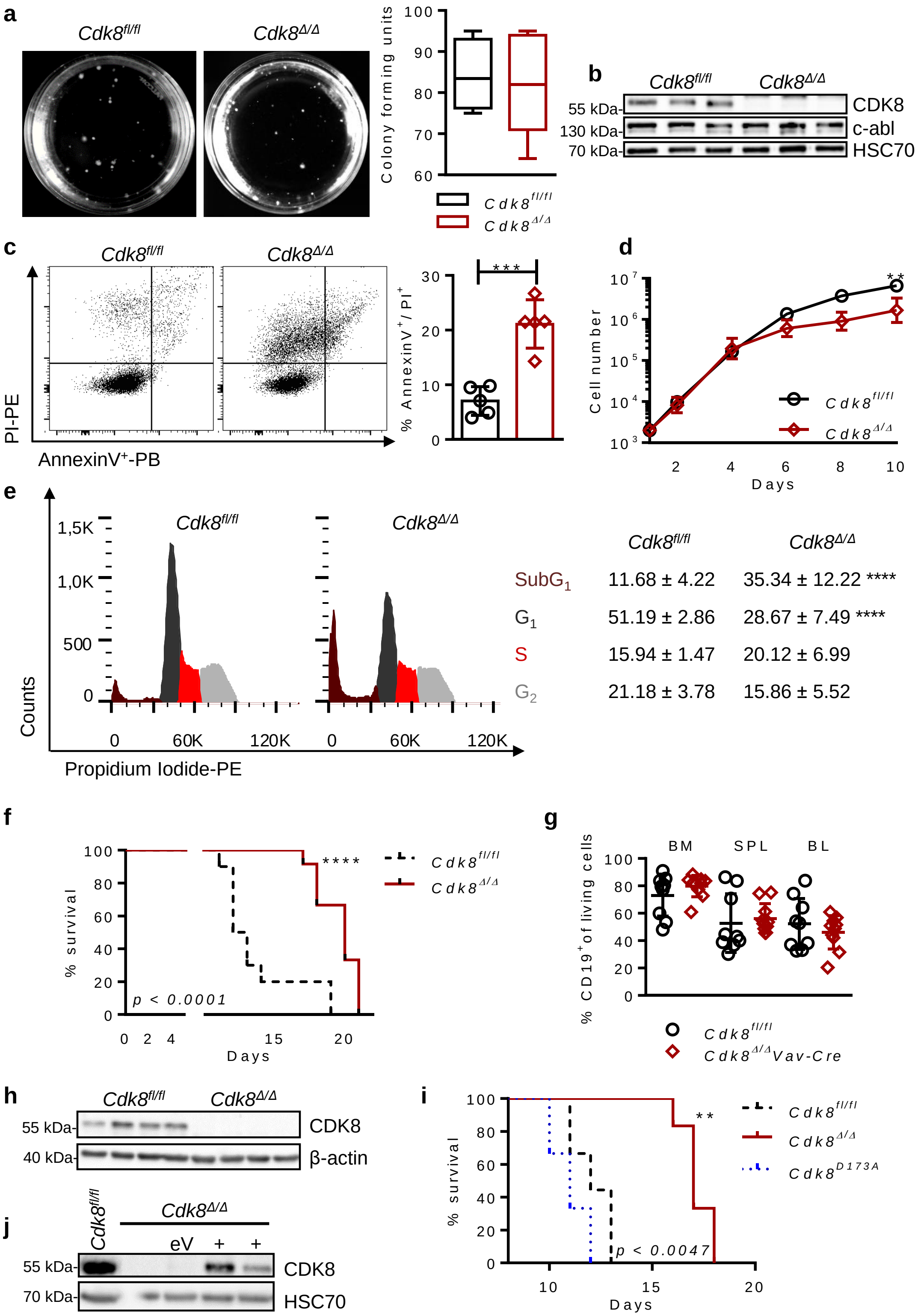
a
Cdk8fl/fl
Cdk8Δ/Δ
Colony forming units
100
90
80
70
60
Cdk8fl/fl
Cdk8Δ/Δ
b
Cdk8fl/fl
Cdk8Δ/Δ
55 kDa-
130 kDa-
70 kDa-
CDK8
c-abl
HSC70
c
Cdk8fl/fl
Cdk8Δ/Δ
PI-PE
AnnexinV+-PB
% AnnexinV+/PI+
30
20
10
0
***
d
Cell number
10 7
10 6
10 5
10 4
10 3
2
4
6
8
10
Days
**
Cdk8fl/fl
Cdk8Δ/Δ
e
Cdk8fl/fl
Cdk8Δ/Δ
1,5K
1,0K
500
0
Counts
0
60K
120K
Propidium Iodide-PE
Cdk8fl/fl
Cdk8Δ/Δ
SubG1 11.68 ± 4.22 35.34 ± 12.22 ****
G1 51.19 ± 2.86 28.67 ± 7.49 ****
S 15.94 ± 1.47 20.12 ± 6.99
G2 21.18 ± 3.78 15.86 ± 5.52
f
% survival
100
80
60
40
20
0
0 2 4
15
20
Days
****
p < 0.0001
Cdk8fl/fl
Cdk8Δ/Δ
g
BM
SPL
BL
% CD19+ of living cells
100
80
60
40
20
0
Cdk8fl/fl
Cdk8Δ/ΔVav-Cre
h
Cdk8fl/fl
Cdk8Δ/Δ
55 kDa-
40 kDa-
CDK8
β-actin
i
% survival
100
80
60
40
20
0
10
15
20
Days
**
p < 0.0047
Cdk8fl/fl
Cdk8Δ/Δ
Cdk8D173A
j
Cdk8fl/fl
Cdk8Δ/Δ
eV
+
+
55 kDa-
70 kDa-
CDK8
HSC70

Supplementary Figure 3: CDK8 is not required for initial v-ABL^{p160+} transformation. **a** v-ABL^{p160}-induced colony formation of $Cdk8^{fl/fl}$ and $Cdk8^{\Delta/\Delta}$ *Vav-Cre* BM cells in growth-factor free methylcellulose. Summary of colony formation assays ($Cdk8^{fl/fl}$ n = 2 and $Cdk8^{\Delta/\Delta}$ *Vav-Cre* n = 4 per genotype in duplicates). Center value represents median, the box 25th to 75th percentiles and whiskers min to max. **b** CDK8 and c-ABL protein levels in v-ABL^{p160+} cell lines. HSC70 served as loading control. **c** Representative FACS profile of an AnnexinV/PI staining of v-ABL^{p160+} $Cdk8^{fl/fl}$ and v-ABL^{p160+} $Cdk8^{\Delta/\Delta}$*Vav-Cre* cell lines and summary of the data (n = 3 per genotype, measured in duplicates). **d** Growth curve of v-ABL^{p160+} $Cdk8^{fl/fl}$ and v-ABL^{p160+} $Cdk8^{\Delta/\Delta}$*Vav-Cre* cell lines. Experiment was performed in triplicates (n = 2 per genotype). (**e**) PI cell cycle staining of v-ABL^{p160+} $Cdk8^{fl/fl}$ and v-ABL^{p160+} $Cdk8^{\Delta/\Delta}$*Vav-Cre* cell lines. Table indicates frequencies of cells in individual phases. **f** V-ABL^{p160+} $Cdk8^{fl/fl}$ and v-ABL^{p160+} $Cdk8^{\Delta/\Delta}$*Vav-Cre* cells were injected intravenously into non-irradiated NSG mice (100000 cells/mouse, n = 10 mice received v-ABL1^{p160+} $Cdk8^{fl/fl}$ and 12 mice v-ABL1^{p160+} $Cdk8^{\Delta/\Delta}$*Vav-Cre* cells*,* 3 independent cell lines per genotype). Survival curves of recipients (median survival of $Cdk8^{fl/fl}$ and $Cdk8^{\Delta/\Delta}$*Vav-Cre* cohorts: 12.5 and 20 days). **g** Summary of CD19^{+} cell distribution in diseased mice. **h** Immunoblotting for CDK8 of ex vivo derived v-ABL^{p160+} $Cdk8^{fl/fl}$ and v-ABL^{p160+} $Cdk8^{\Delta/\Delta}$*Vav-Cre* cells. Levels of β-actin served as loading controls. **I** v-ABL^{p160+} $Cdk8^{D173A}$, v-ABL^{p160+} $Cdk8^{fl/fl}$ and v-ABL^{p160+} $Cdk8^{\Delta/\Delta}$*Vav-Cre* cells were injected intravenously into non-irradiated NSG mice (100000 cells/mouse, n = 9 mice received v-ABL1^{p160+} $Cdk8^{fl/fl}$ and 6 mice v-ABL^{p160+} $Cdk8^{D173A}$ or v-ABL1^{p160+} $Cdk8^{\Delta/\Delta}$*Vav-Cre* cells*,* 2 independent cell lines per genotype). Survival curves of recipients are depicted (median survival of Cdk8^{D173A}, $Cdk8^{fl/fl}$ and $Cdk8^{\Delta/\Delta}$*Vav-Cre* cohorts: 11, 12 and 17 days). **j** Immunoblotting for CDK8 of v-ABL^{p160+} $Cdk8^{fl/fl}$, v-ABL^{p160+} $Cdk8^{\Delta/\Delta}$*Vav-Cre,* v-ABL^{p160+} $Cdk8^{\Delta/\Delta}$*Vav-Cre* + empty Vector *(eV)* and v-ABL^{p160+} $Cdk8^{\Delta/\Delta}$*Vav-Cre* reconstituted with CDK8 kinase-dead mutant $Cdk8^{D173A}$ cells. HSC70 served as loading control. Levels of significance were calculated using **d**, **g** (BM) Mann-Whitney, **a**, **c**, **e** and **g** (SPL, BL) unpaired *t*-test and **f**, **i** long-rank test, data represents means ± SD (**p < 0.01; ***p<0.001; ****p<0.0001). Source data are provided as a Source Data file

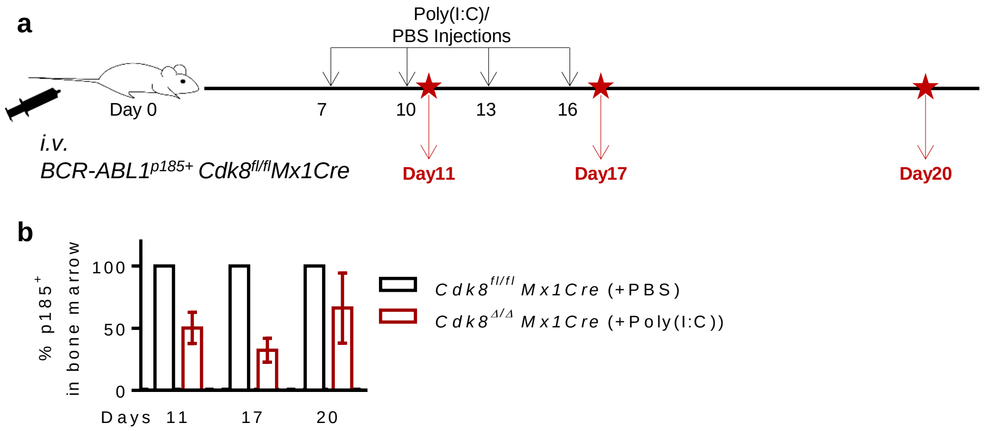

Supplementary Figure 4: CDK8 is required for maintenance of BCR-ABL1^{p185+} **a** Experimental setup of in vivo time course experiment. Non-irradiated NSG mice received BCR-ABL1^{p185+} *Cdk8$^{fl/fl}$ Mx1Cre* cell lines and were analysed at the indicated time point for BCR-ABL1^{p185+} cell in the BM (2500 cells/mouse, n = 3 received PBS as vehicle control and n = 9 received intraperitoneal poly (I:C) (200 µg) injections, 3 independent cell lines were used). **b** Bar diagram depicts percentages of BCR-ABL1^{p185+} cells in the bone marrow on days 11, 17 and 20. Data represents means ± SD

a

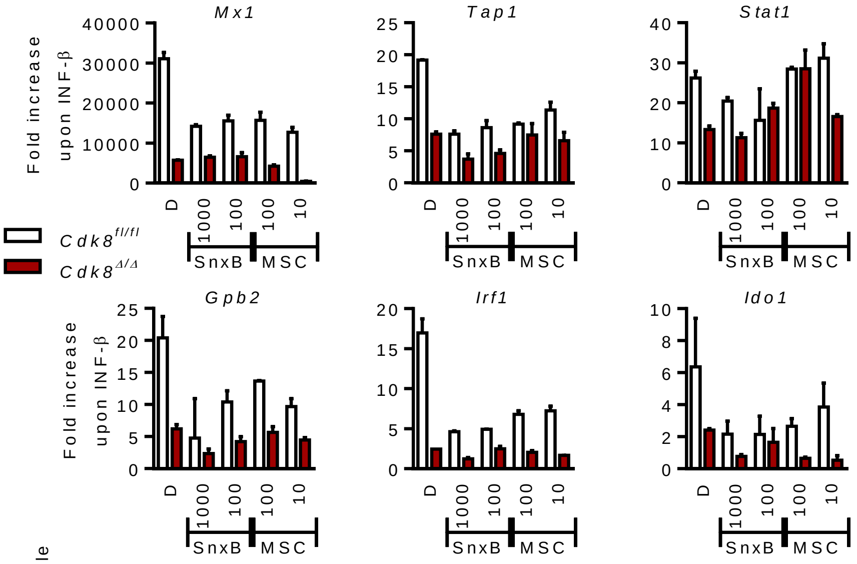

b

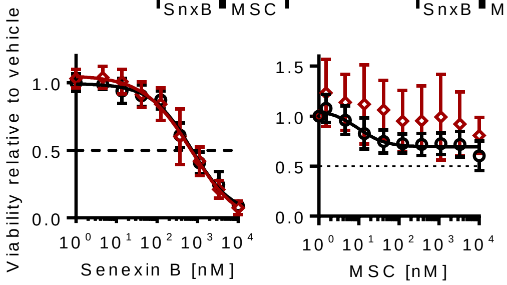

IC$_{50}$ [nM]	*Cdk8$^{fl/fl}$*	*Cdk8$^{Δ/Δ}$*
SnxB	724	718

c

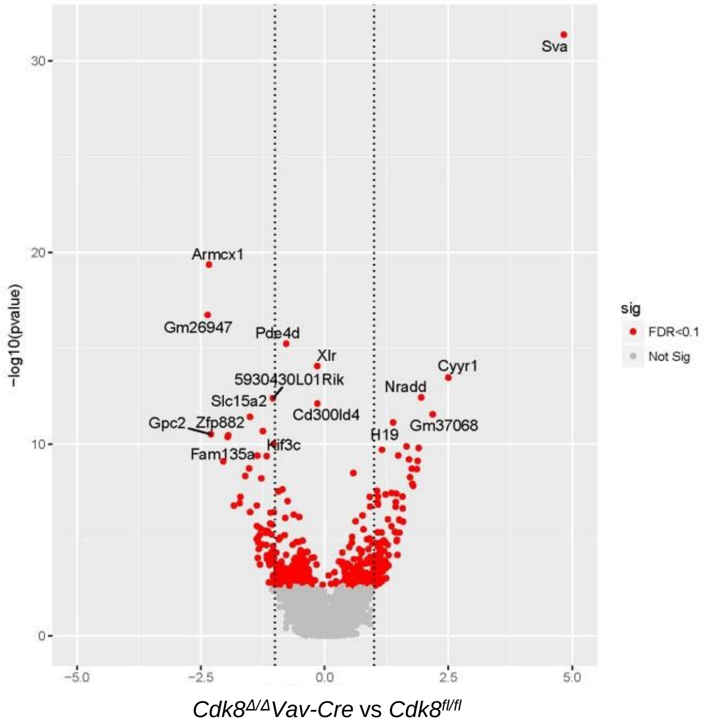

Supplementary Figure 5: CDK8 kinase-inhibition fails to mimic the effects of *Cdk8* deletion in BCR-ABL1^{p185+} cell lines. **a** RT-qPCRs of STAT1 target genes *Mx1*, *Stat1*, *Tap1*, *Gpb2*, *Irf1* and *Ido1*. BCR-ABL1^{p185+} *Cdk8$^{fl/fl}$* were incubated 48 hours with 1000 nM or 100 nM SenexinB (SnxB) or 100 nM or 10nM MSC and stimulated 24 hours with interferon-β. Fold increase is relative to unstimulated gene expression. Bars depict mean ± SD. **b** Dose-response curves of BCR-ABL1^{p185+} *Cdk8$^{fl/fl}$* and BCR-ABL1^{p185+} *Cdk8$^{Δ/Δ}$Vav-Cre* cell lines (sum of MSC n = 2 and SnxB n = 5 cell lines per genotype, measured in quadruplicates (MSC) or triplicates (SnxB); mean ± SD). IC_{50}´s for Senexin B of BCR-ABL1^{p185+} *Cdk8$^{fl/fl}$ and* BCR-ABL1^{p185+} *Cdk8$^{Δ/Δ}$Vav-Cre* are listed in the table. **c** Volcano Blot of the 159 differentially expressed genes of BCR-ABL1^{p185+} *Cdk8$^{Δ/Δ}$Vav-Cre* vs BCR-ABL1^{p185+} *Cdk8$^{fl/fl}$* cell lines. Source data are provided as a Source Data file

a

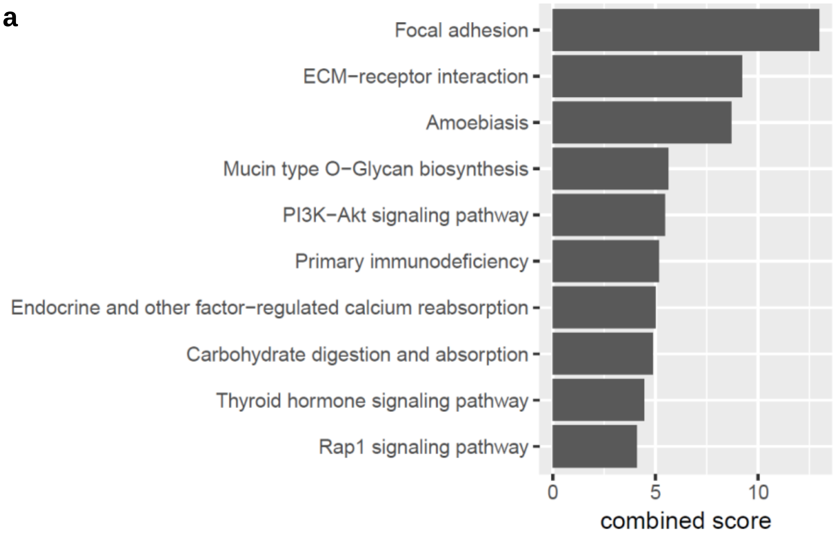

b

Gene	FC cp Cdk8$^{fl/fl}$
Tpfi	0,007 ± 0.008
Armcx1	0,087 ± 0.156
Bcl2	0,142 ± 0.161
Ptk2	0,160 ± 0.242
Kif3c	0,215 ± 0.167
Id1	0,281 ± 0.313
Epas1	0,314 ± 0.326
Socs5	0,319 ± 0.322
Sox8	0,355 ± 0.130
Nfκb2	0,374 ± 0.200
Tnfα	0,447 ± 0.169
Mcl1	0,486 ± 0.163
Nfkb1	0,494 ± 0.256
Itgd2	0,504 ± 0.296
Bax1	0,508 ± 0.236
p21	0,536 ± 0.147
Notch1	0,741 ± 0.239
Atm	0,773 ± 0.230

c

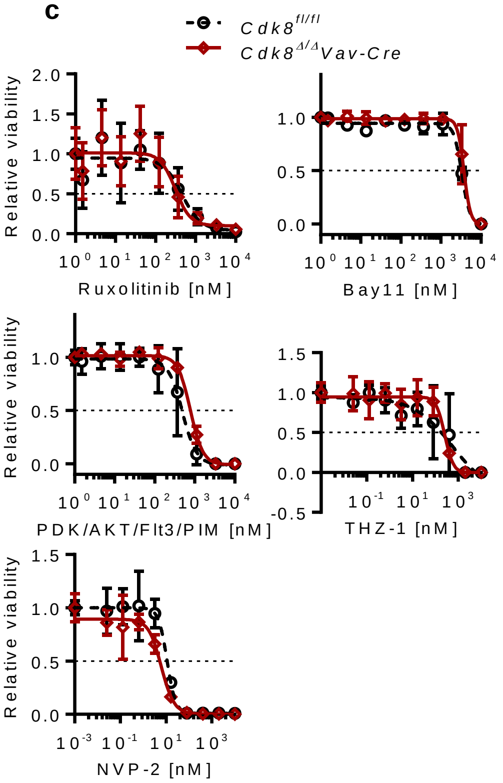

Supplementary Figure 6: CDK8 regulates the mTOR pathway. **a** Top 10 enriched KEGG pathways of genes down-regulated in BCR-ABL1^{p185+} *Cdk8$^{Δ/Δ}$Vav-Cre* vs. BCR-ABL1^{p185+} *Cdk8$^{fl/fl}$* cell lines. Top 10 KEGG pathways obtained by Enrichr ranked by the combined score (log (Fisher exact test p-value) * z-score). For p-values and z-scores, see Supplementary Table 2. **b** Table shows fold changes in gene expression BCR-ABL1^{p185+} *Cdk8$^{Δ/Δ}$Vav-Cre* compare to BCR-ABL1^{p185+} *Cdk8$^{fl/fl}$* of a set of genes that were found to be differentially expressed (RNA-seq) by qRT-PCR. Values display mean ± SD of n = 4 per genotype measured in triplicates. **c** Dose-response curves for Ruxolitinib, Bay11, PDK/AKT/Flt3/PIM, THZ-1 and NVP-2; mean ± SD. Source data are provided as a Source Data file

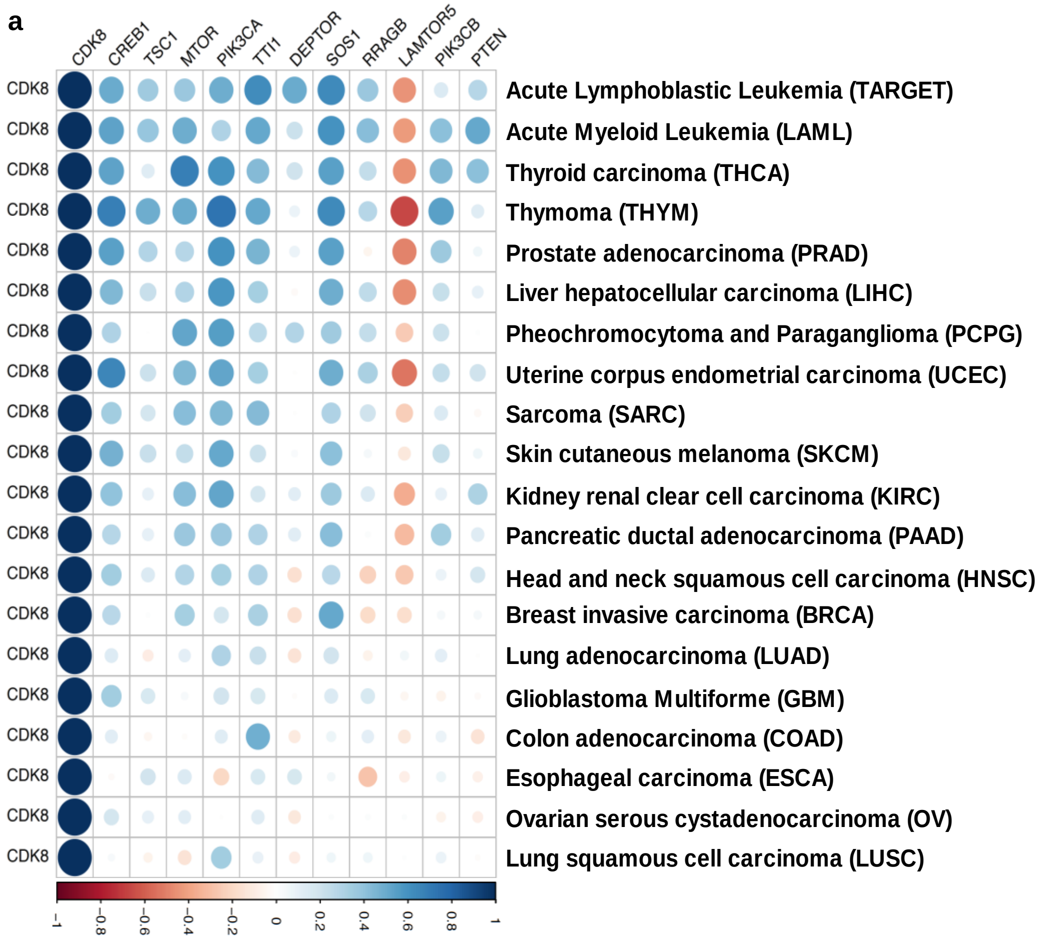

Supplementary Figure 7: Co-expression matrix of *Cdk8* and members of the mTOR pathway. **a** Co-expression of *Cdk8* and members of mTOR/PI3K signaling pathways in a pediatric ALL dataset (TARGET cohort, RNA-seq) and in 19 TCGA RNA-seq datasets. The scale indicates Spearman correlation coefficients ranging from −1 (red) to 1 (blue).

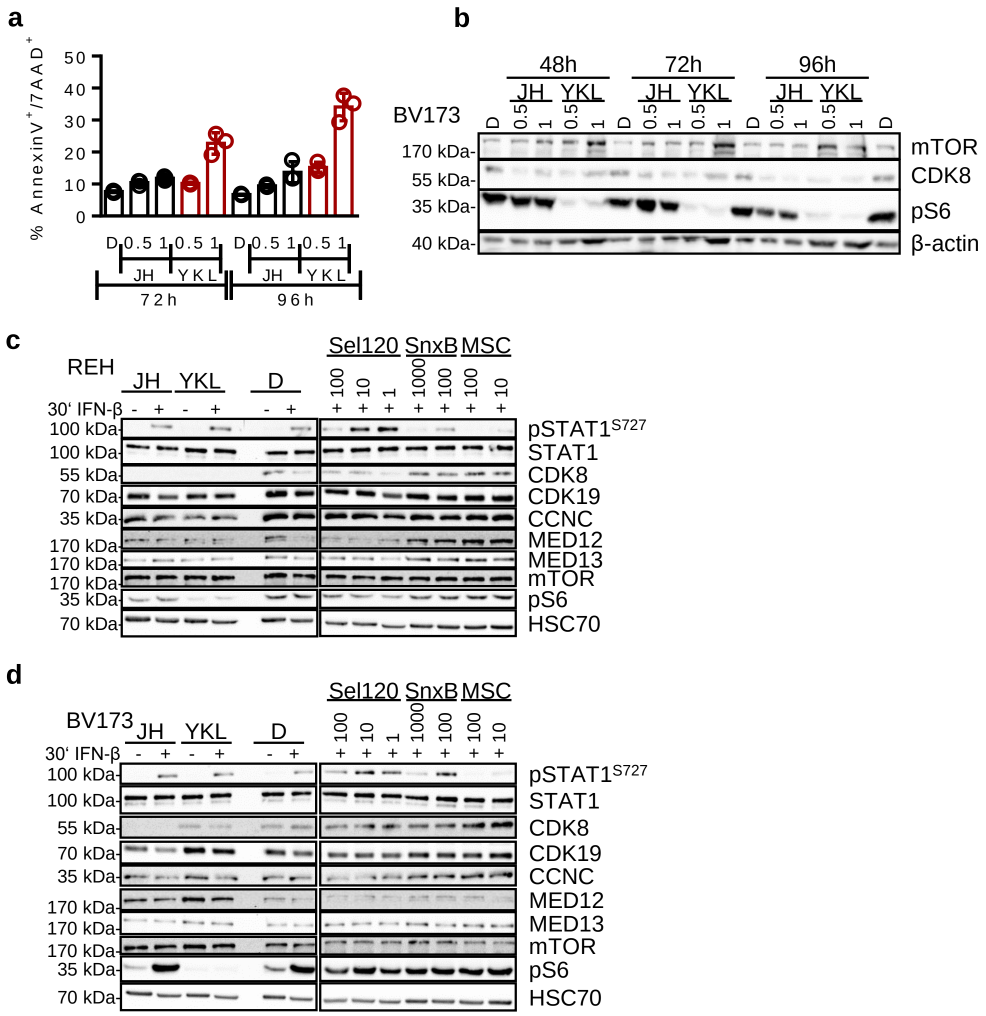

Supplementary Figure 8: Chemical CDK8 degradation cooperates with mTOR inhibition. **a** AnnexinV/7AAD staining of BV173 cells incubated with 0.5 µM and 1 µM of JH-XI-10-02 or YKL-06-101 for 72 and 96 hours. DMSO was used as vehicle control (D: DMSO, n = 3). **b** Immunoblotting of mTOR, CDK8 and pS6$^{S240/244}$ of BV173 cell line incubated with 0.5 µM and 1 µM of JH-XI-10-02 or YKL-06-101 for 48, 72 and 96 hours. DMSO (indicated as D) served as vehicle control and β-actin as loading control. REH **c** or BV173 **d** incubated with 1 µM of JH-XI-10-02 or YKL-06-101 or indicated concentrations [nM] of Sel120, SnxB or MSC for 48 hours. Plus (+) indicates samples treated 30 min with interferon-β (IFN-β) before harvesting. Blots were probed for pSTAT1^{S727}, total STAT1, CDK8, CDK19, CCNC, MED12, MED13, mTOR and pS6$^{S240/244}$. DMSO (indicated as D) served as vehicle control and HSC as loading control. Levels of significance were calculated using Kruskal-Wallis test followed by Dunn's test, data represents means ± SD. Source data are provided as a Source Data file

Supplementary Tables

Supplementary Table 1: Genes set enrichment analysis.

Gene set enrichment analysis – Hallmarks					
Gene sets	ES	NES	NOM p-val	FDR q-val	FWER p-val
Apical surface	-0.50	-1.68	0.000	0.010	0.004
TNFα signaling via NFκB	-0.42	-1.66	0.000	0.014	0.010
UV response	-0.36	-1.41	0.004	0.132	0.147
E2F targets	-0.36	-1.40	0.000	0.099	0.147
KRAS signaling	-0.35	-1.39	0.004	0.096	0.173
G2M Checkpoint	-0.35	-1.37	0.004	0.093	0.197
MTORC1 signaling	-0.33	-1.34	0.000	0.109	0.253
PI3K AKT MTOR signaling	-0.36	-1.34	0.022	0.097	0.258
Inflammatory response	-0.34	-1.33	0.007	0.100	0.296
Cholesterol homeostasis	-0.38	-1.32	0.041	0.091	0.298
Estrogen response early	-0.33	-1.31	0.008	0.092	0.327
IL2 STAT5 signaling	-0.31	-1.27	0.004	0.128	0.456
Unfolded protein response	-0.34	-1.26	0.037	0.130	0.488
Heme metabolism	-0.31	-1.24	0.017	0.139	0.541
Spermatogenesis	-0.32	-1.22	0.034	0.161	0.623
Allograft rejection	-0.30	-1.21	0.037	0.178	0.684
Oxidative phosphorylation	-0.30	-1.20	0.043	0.174	0.703
Hedgehog signaling	-0.36	-1.14	0.243	0.285	0.874
p53 pathway	-0.36	-1.14	0.243	0.285	0.874
Angiogenesis	-0.36	-1.13	0.243	0.284	0.900
IL6 JAK-STAT3 signaling	-0.32	-1.11	0.248	0.317	0.929
Hypoxia	-0.28	-1.10	0.159	0.33	0.945
Apoptosis	-0.28	-1.10	0.195	0.325	0.950
Interferon gamma response	-0.28	-1.10	0.129	0.313	0.951
Estrogen response late	-0.27	-1.07	0.208	0.367	0.974
Adipogenesis	-0.27	-1.07	0.207	0.360	0.975
Pancreas beta cells	-0.35	-1.06	0.325	0.377	0.982
Reactive oxygen species pathway	-0.31	-1.06	0.316	0.372	0.985
Apical junction	-0.26	-1.05	0.280	0.402	0.994

ES: enrichment score; NES: normalized enrichment score; NOM p-val: nominal p-value; FDR q-val: False discovery rate q-value; FWER p-val: familywise-error rate p-value

Supplementary Table 2: EnrichR pathway analysis.

Term	Overlap	P-value	Z-score	Combined score	Genes
Focal adhesion	7/202	0,0011	-1,922	12,9539	LAMA5;ACTN1;SPP1;PTK2;THBS3;IGF1R;ITGA9
ECM-receptor interaction	4/82	0,0041	-1,6821	9,2221	LAMA5;SPP1;THBS3;ITGA9
Amaeoiasis	4/100	0,0083	-1,8183	8,6999	LAMA5;ACTN1;PLCB2;PTK2
Mucin type O-Glycan biosynthesis	2/31	0,0251	-1,524	5,6147	GALNT6;GALNT10
PI3K-Akt signaling pathway	6/341	0,0552	-1,8856	5,4591	LAMA5;SPP1;PTK2;THBS3;IGF1R;ITGA9
Primary immunodeficiency	2/37	0,0348	-1,538	5,1612	RAG2;RAG1
Edocrine and other factor-regulated calcium reabsorption	2/47	0,0537	-1,7135	5,0078	ATP1A3;PLCB2
Carbohydrate digestion and absorption	2/45	0,0497	-1,625	4,8755	ATP1A3;PLCB2
Thyroid hormone signaling pathway	3/118	0,0679	-1,6469	4,4274	RCAN1;ATP1A3;PLCB2
Rap1 signaling pathway	4/211	0,088	-1,685	4,0946	ID1;PLCB2;RAP1GAP;IGF1R

Results from EnrichR pathway analysis using significant downregulated genes (fold change > 2, padjust < 0.1) between BCR-ABL1^{p185+} *Cdk8*$^{\Delta/\Delta}$*Vav-Cre* vs. BCR-ABL1^{p185+} *Cdk8*$^{fl/fl}$ cell lines.

Supplementary Table 3: Patient information.

Pat. No No#	Gender (f/m)	Age (years)	Diagnosis	WBC (g/l)	Blasts (%) BM	Blasts (%) PB	BCR-ABL1 (variant)	sample	JH-XI-10-02 IC_{50}	YKL-06-101 IC_{50}
#1	f	41	T-ALL	2.66	94	n.a.	-	BM	> 10 µM	> 10 µM
#3	m	72	c-ALL	2.42	80-85	18	-	BM	> 10 µM	0.9 µM
#4	m	69	prä B-ALL	17.04	71	46	+(p190)	BM	> 10 µM	> 10 µM
#6	f	22	T-ALL	56.65	90	61	-	PB	> 10 µM	0.1 µM
#7	m	333	T-ALL	14.53	40	n.a.	-	BM	> 10 µM	> 10 µM
#8	f	71	c-ALL	69.58	85	73	-	PB	> 10 µM	> 10 µM
#9	f	73	c-ALL	11.98	90-95	23	-	BM	> 10 µM	9 µM
#11	m	51	c-ALL	2.79	88	19	-	BM	> 10 µM	0.4 µM
#12	f	76	pro B-ALL	14.88	95	67	-	PB	> 10 µM	> 10 µM
#13	m	36	T-ALL	6.44	22	n.a.	-	BM	> 10 µM	0.3 µM
#14	m	39	T-ALL	19.83	80	56	-	BM	> 10 µM	> 10 µM
#15	f	50	prä B-ALL	94.01	80	92	-	BM	> 10 µM	10 µM

Pat. No: patient number; WBC: white blood cell count; f: female, m: male; G/l: 10^9 cells per liter; BM: bone marrow, PB: peripheral blood; -: negative for BCR-ABL1; +: positive for BCR-ABL1, n.a.: not available

Supplementary Methods

Supplementary Table 4: Sequences of shRNAs used in this study

Ren. 713
TGCTGTTGACAGTGAGCGCAGGAATTATAATGCTTATCTATAGTGAAGCCACAGATGTATAGATAAGCATTATAATTCCTATGCCTACTGCCTCGGA
Myc.1888
TGCTGTTGACAGTGAGCGAGAAACGACGAGAACAGTTGAATAGTGAAGCCACAGATGTATTCAACTGTTCTCGTCGTTTCCTGCCTACTGCCTCGGA
Cdk6.897
TGCTGTTGACAGTGAGCGACTGACGTTTAATCCAGCTAAATAGTGAAGCCACAGATGTATTTAGCTGGATTAAACGTCAGGTGCCTACTGCCTCGGA
Cdk7.1145
TGCTGTTGACAGTGAGCGCCAGTTTCACTGCTGAAATAAATAGTGAAGCCACAGATGTATTTATTTCAGCAGTGAAACTGTTGCCTACTGCCTCGGA
Cdk8.2547
TGCTGTTGACAGTGAGCGCAAACAAGGTGTTATGTAATAATAGTGAAGCCACAGATGTATTATTACATAACACCTTGTTTTTGCCTACTGCCTCGGA
Cdk9.2872
TGCTGTTGACAGTGAGCGATGGCATGAAGACAAACTAATATAGTGAAGCCACAGATGTATATTAGTTTGTCTTCATGCCACTGCCTACTGCCTCGGA
Ccnc.574
TGCTGTTGACAGTGAGCGAAGGATGAATCATATACTAGAATAGTGAAGCCACAGATGTATTCTAGTATATGATTCATCCTGTGCCTACTGCCTCGGA
Cdk19.1560
TGCTGTTGACAGTGAGCGAAGGGTGGTATTTGTGTTACAATAGTGAAGCCACAGATGTATTGTAACACAAATACCACCCTGTGCCTACTGCCTCGGA
Med12.3320
TGCTGTTGACAGTGAGCGCCCGCTATAGCTTTGTGTGCAATAGTGAAGCCACAGATGTATTGCACACAAAGCTATAGCGGTTGCCTACTGCCTCGGA
Med13.9367
TGCTGTTGACAGTGAGCGAACCAAGTGTACTTATATGTAATAGTGAAGCCACAGATGTATTACATATAAGTACACTTGGTGTGCCTACTGCCTCGGA

Supplementary Table 5: Primers and PCR program used for genotyping.

Genotype	PCR program			Forward primer (F) (5'-3')<br>Reverse primer (R) (5'-3')	Product size (bp)
$Cdk8^{fl/fl}$<br>$Cdk8^{\Delta/\Delta}$	5'	95°C	x1	L3F2: ACCTTCCCCCTGTCTCAGACAGTCCTT<br>L2R: AGGAAAACGACAATGGAAGCAGCAGC<br>Ef2(Δ0):CGTAGGTAGCAATCTGGTCGGGGT	600 (Δ)<br>499 (fl)<br>446 (wt)
	30''<br>40''<br>40''	95°C<br>60°C<br>72°C	x35		
	5'	72°C	x1		
Mx1Cre	5'	95°C	x1	F: CGGTCGATGCAACGAGTGATGAGG<br>R: CCAGAGACGGAAATCCATCGCTCG	700
	30''<br>40''<br>40''	95°C<br>57°C<br>72°C	x35		
	5'	72°C	x1		
VavCre	5'	95°C	x1	F: AGATGCCAGGACATCAGGAACCTG<br>R: ATCAGCCACACCAGACACAGAGATC	236
	30''<br>40''<br>40''	95°C<br>60°C<br>72°C	x35		
	5'	72°C	x1		

Supplementary Table 6: Flow cytometry antibodies used in this study (working dilution 1:100)

Name	Clone	Conjugate	Company	Cat. no.
BP-1	6C3	Biotin	eBioscience	13-5891
CD11b (Mac1)	M1/70	eFluor450<br>PerCP-Cy5.5	eBioscience	48-0112<br>45-0112
CD127 (IL-7R alpha)	A7R34	Biotin	eBioscience	13-1271
CD135 (Flt3)	A2F10	Biotin	eBioscience	13-1351
CD150 (SLAM)	TC15-12F12.2	APC	BioLegend	115910
CD16/CD32 Fc gamma RII/III	93	PE	eBioscience	12-0161
CD19	eBio1D3	APC-Cy7<br>eFluor780<br>eFluor450	BD<br>eBiosciennce	557655<br>47-0193<br>48-0193
CD3	17A2	eFluor450	eBioscience	48-0032
CD34	RAM34	FITC<br>APC	BD	553733<br>560230
CD4	GK1.5	PE-Cy7<br>FITC	eBioscience<br>BD	25-0041<br>553729

CD43	S7	PE	BD	553271
CD45.1	A20	PE	eBioscience	12-0453
CD45.2	104	APC-eFluor780<br>eFluor450	eBioscience	47-0454<br>48-0454
CD45R (B22)	RA3-6B2	eFluor450<br>PerCP-Cy5.5	eBioscience	48-0452<br>45-0452
CD48	HM48-1	PE	eBioscience	12-0481
CD8a	53-6.7	APC<br>APC	ImmoTool<br>BD	22150086<br>553035
c-kit (CD117)	2B8	PerCP-Cy5.5	eBioscience	15-1171
Gr1 (Ly6G/Ly6C)	RB6-8C5	eFluor450<br>FITC<br>APC<br>APC-eFluor780	eBioscience<br>BD<br>eBioscience	48-5931<br>553127<br>17-8931<br>47-5931
IgD	11-26	APC	eBioscience	17-5993
IgM	11/41	FITC	eBioscience	11-5790
Sca-1 (Ly6A/E)	D7	PE-Cy7	eBioscience	25-5981
Streptavidin		APC<br>APC-eFluor780<br>eFluor450	eBioscience	14-4317<br>47-4317<br>48-4317
Ter119	TER-119	eFluor450<br>PE	eBioscience	48-5921<br>12-5921

Supplementary Table 7: Western Blot antibodies used in this study.

Antigen	Source	Dilution	Company	Cat. no.
4E-BP1 (53H11)	rabbit	1:1.000	Cell Signaling	CS#9644
Akt (40D4)	mouse	1:1.000	Cell Signaling	CS#2920
c-abl (K-12)	rabbit	1:1000	Santa Cruz	sc-131
CDK19	rabbit	1:500	Sigma-Aldrich	HPA007053
CDK6 (H96)	rabbit	1:1.000	Santa Cruz	sc-7180
CDK7	rabbit	1:1.000	Cell Signaling	CS#2090
CDK8	rabbit	1:1000	ThermoScientific	PA1-21780

CDK8 (G398)	rabbit	1:1.000	Cell signaling	CS#4101
CDK9 (C12F7)	rabbit	1:1.000	Cell Signaling	CS#2316
c-Myc (C19)		1:1000	Santa Cruz	Sc-788
CyclinC (T-19)	rabbit	1:1000	Santa Cruz	sc-1061
HSC-70	mouse	1:1.000	Santa Cruz	sc-7298
MED12 (D9K5J)	rabbit	1:1.000	Cell Signaling	CS#14360
MED13	rabbit	1:500	ThermoScientific	#PA5-79654
Mouse IgG-HRP		1:10.000	Cell signaling	CS#7076
mTOR (30)	mouse	1:1.000	Santa Cruz	sc-517464
p4E-BP1$^{T37/46}$ (236B4)	rabbit	1:1.000	Cell Signaling	CS#2855
pAKT^{S473} (D9E)	rabbit	1:1000	Cell Signaling	CS#4060
pS6$^{S240/244}$ (D68F8)	rabbit	1:1.000	Cell Signaling	CS#5364
pSTAT1^{S727}	rabbit	1:1.000	Cell Signaling	CS#9177
pSTAT5^{S725}	rabbit	1:1.000	Eurogentec	customized
Rabbit IgG-HRP		1:5.000	Cell Signaling	CS#7074
S6 (54D2)	mouse	1:1.1000	Cell Signaling	CS#2317
STAT1	mouse	1:1.000	Santa Cruz	sc-592
STAT5A/B C-17	rabbit	1:1.000	Santa Cruz	sc-835
β-actin	mouse	1:1.000	Santa Cruz	sc-69879

Supplementary Table 8: qRT-PCR primers used in this study

mEpas1	Forward<br>Reverse	TGACGATGTGGCTGTAACTG<br>GAGGGTTCATCGAAGTTCTGG
mBcl2	Forward<br>Reverse	GATGACTGAGTACCTGAACCG<br>CAGAGACAGCCAGGAGAAATC
mKif3c	Forward<br>Reverse	CAGGACCAACACGAAGAGTAC<br>GAAAAGGCGGTTCATGATCTTG
mSocs5	Forward<br>Reverse	TTTGGTAGAACTCGAAGCGG<br>GAAAACACAAACCCACCGTG
mId1	Forward<br>Reverse	GCTGAACTCGGAGTCTGAAG<br>GCCTCAGCGACACAAGATG
mSox8	Forward<br>Reverse	CATCTCCATAACGCAGAGCTC<br>TGGCTGGTATTTGTAATCTGGG
mPtk2	Forward	AGTTTGACCATCCTCACATCG

	Reverse	TCAAAGATGCCAGGTCCAAG
mArmcx1	Forward	GGTGCCTGCTACTGTGTATAC
	Reverse	CCCTACCCCAACATTAGTCTTAAG
mTfpi	Forward	CCCAGTGAATGAGGTACAGATG
	Reverse	GTTGAAGACACCAGGGACG
mAtm	Forward	GATCCTTCCCACTCCAGAAAC
	Reverse	ACTCCGCATAACTTCCATCG
mNfkb1	Forward	AAGACAAGGAGCAGGACATG
	Reverse	AGCAACATCTTCACATCCCC
mNfkb2	Forward	CACCCATCTAGTCACCAAGC
	Reverse	TCAGCACCAGCCTTTAGAAG
mTnfalpha	Forward	CCCTCACACTCAGATCATCTTCTC
	Reverse	GTCTTTGAGATCCATGCCGTTG
mMx1	Forward	GACTACCACTGAGATGACCCAGC
	Reverse	ATTTCCTCCCCAAATGTTTTCA
mSTAT1	Forward	CACATTCACATGGGTGGAAC
	Reverse	TCTGGTGCTTCCTTTGGTCT
mIdo1	Forward	GAGGATGCGTGACTTTGTGGAC
	Reverse	CATACAGCAGACCTTCTGGCAG
mIrf1	Forward	CGGACACTTTCTCTGATGGA
	Reverse	CGGAGGTGGAAGGCATGGGTGA
mGpb2	Forward	TGCTAAACTTCGGGAACAGG
	Reverse	GAGCTTGGCAGAGAGGTTTG
mTap1	Forward	CTGGCAACCAGCTACGGGT
	Reverse	TGAGAAAGAGGATGTGGTGGG
mMcl1	Forward	CGCAACCACGAGACGGCCTT
	Reverse	ACTCCACAAACCCATCCCAGCCT
mNotch	Forward	CAATGACCCCTGGAAGAACTGC
	Reverse	GCCATCCCACTCACATTCGG
mp21	Forward	GAACATCTCAGGGCCGAAA
	Reverse	ATCTGCGCTTGGAGTGATAG
mBax	Forward	GGAGATGAACTGGACAGCAA
	Reverse	AAGTTGCCATCAGCAAACAT
mItgb2	Forward	ATGCACCAAGTACAAAGTCAGC
	Reverse	TTGGTCGAACTCAGGATTAGC
mUbe2d2a	Forward	AGGTCCTGTTGGAGATGATATGTT
	Reverse	TTGGGAAATGAATTGTCAAGAAA
mβ-actin	Forward	CTCTGGCTCCTAGCACCATGAAGA
	Reverse	GTAAAACGCAGCTCAGTAACAGTCC
mHprt	Forward	GGATTTGAATCACGTTTGTGTCAT
	Reverse	ACACCTGCTAATTTTACTGGCAA

Supplementary Note 1: Synthetic Scheme of YKL-06-101 degrader

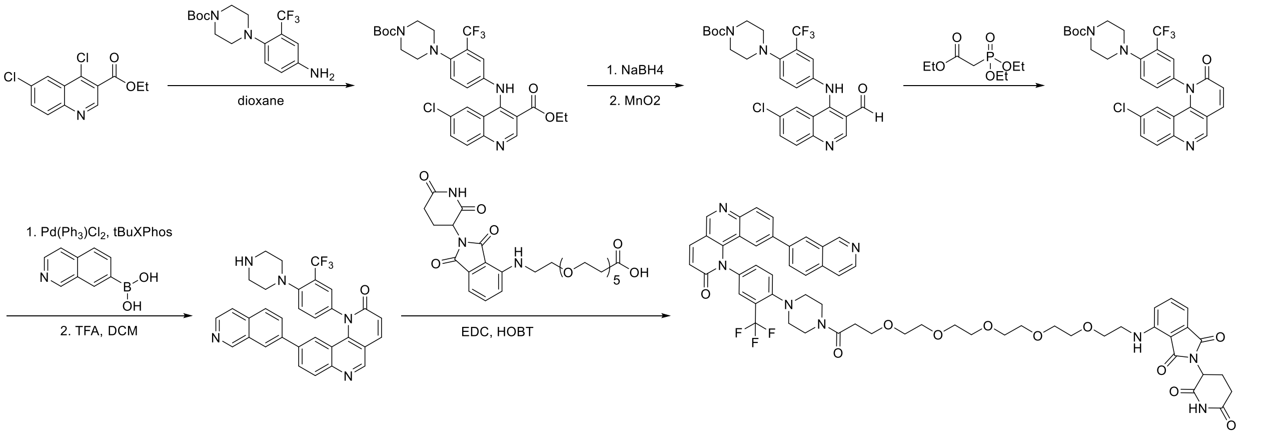

Step 1: Ethyl 4-((4-(4-(tert-butoxycarbonyl)piperazin-1-yl)-3-(trifluoromethyl)phenyl)amino)-6-chloroquinoline-3-carboxylate

Ethyl 4,6-dichloroquinoline-3-carboxylate (569 mg, 2.1 mmol), tert-butyl 4-(4-amino-2-(trifluoromethyl)phenyl)piperazine-1-carboxylate (727 mg, 2.1 mmol), and DIEA (258 mg, 0.35 mL, 2 mmol) were mixed in dioxane (10 mL). The mixture was stirred at 100 °C overnight. The reaction was concentrated and purified by flash column chromatography on silica gel (0-70% EtOAc in hexane) to give the title compound as a yellow solid (465 mg, 38%). LCMS: m/z 579.2 [M+1].

Step 2: tert-butyl 4-(4-((6-chloro-3-formylquinolin-4-yl)amino)-2-(trifluoromethyl)phenyl)piperazine-1-carboxylate

To a solution of ethyl 4-((4-(4-(tert-butoxycarbonyl)piperazin-1-yl)-3-(trifluoromethyl)phenyl)amino)-6-chloroquinoline-3-carboxylate (465 mg, 0.8 mmol) in EtOH (10 mL) was add NaBH4 (304 mg, 8.0 mmol) slowly. The mixture was stirred at rt overnight. The reaction was concentrated and extracted with EtOAc from H2O, washed with brine, dried (Na2SO4), and concentrated. The residue was mixed in DCM (10 mL) and MnO2 (2 g) was added. The mixture was stirred at room temparature overnight. The mixture was filtered through Celite and concentrated. The residue was put into next step directly.

Step 3: tert-butyl 4-(4-(9-chloro-2-oxobenzo[h][1,6]naphthyridin-1(2H)-yl)-2-(trifluoromethyl)phenyl)piperazine-1-carboxylate

The tert-butyl 4-(4-((6-chloro-3-formylquinolin-4-yl)amino)-2-(trifluoromethyl)phenyl)piperazine-1-carboxylate (crude from step 2) was dissolved in EtOH (10 mL) and triethyl phosphonoacetate (362 mg, 1.6 mmol) was added, followed by K2CO3 (331 mg, 2.4 mmol). The mixture was stirred at 100 °C overnight. The reaction was concentrated and purified by flash column chromatography on silica gel (100% EtOAc) to give the title compound as a yellow solid (138 mg, 31% over three steps). LCMS: m/z 559.2 [M+1].

Step 4: 9-(isoquinolin-7-yl)-1-(4-(piperazin-1-yl)-3-(trifluoromethyl)phenyl)benzo[h][1,6]naphthyridin-2(1H)-one

To a sealed tube was added tert-butyl 4-(4-(9-chloro-2-oxobenzo[h][1,6]naphthyridin-1(2H)-yl)-2-(trifluoromethyl)phenyl)piperazine-1-carboxylate (138 mg, 0.25 mmol), isoquinolin-7-ylboronic acid (86.5 mg, 0.5 mmol), tBuXPhos (10.6 mg, 0.025 mmol), dioxane (6 mL) and Sat. Na2CO3 aq. (1.5 mL). The mixture was stirred at 80 °C overnight. The mixture was filtered and concentrated. The residue was dissolved in DCM (1 mL) and TFA (1 mL) and stirred at room temperature for 1 h. The reaction was

concentrated and purified by flash column chromatography on silica gel (0-10% MeOH in DCM) to give the title compound as a yellow solid (131 mg, 95% over two steps). LCMS: m/z 552.2 [M+1].

Step 5: 2-(2,6-dioxopiperidin-3-yl)-4-((18-(4-(4-(9-(isoquinolin-7-yl)-2-oxobenzo[h][1,6]naphthyridin-1(2H)-yl)-2-(trifluoromethyl)phenyl)piperazin-1-yl)-18-oxo-3,6,9,12,15-pentaoxaoctadecyl)amino)isoindoline-1,3-dione

To a solution of 9-(isoquinolin-7-yl)-1-(4-(piperazin-1-yl)-3-(trifluoromethyl)phenyl)benzo[h][1,6]naphthyridin-2(1H)-one (8.3 mg, 0.015 mmol) and 1-((2-(2,6-dioxopiperidin-3-yl)-1,3-dioxoisoindolin-4-yl)amino)-3,6,9,12,15-pentaoxaoctadecan-18-oic acid (11 mg, 0.015 mmol) in DMF (0.5 mL) were added EDC (3.5 mg, 0.018 mmol), HOBt (2.6 mg, 0.02 mmol) and TEA (7.6 mg, 10 µL, 0.075 mmol). The mixture was stirred at room temperature overnight. The mixture was purified by reverse phase HPLC (0-100% MeOH in H2O) to give the title compound as a yellow solid (7.7 mg, 47%). 1HNMR (500 MHz, DMSO-d6) δ 11.02 (s, 1H), 9.48 (s, 1H), 9.17 (s, 1H), 8.58 (d, J=6.0 Hz, 1H), 8.29 (d, J=9.5Hz, 1H), 8.21 - 8.13 (m, 3H), 8.09 (dd, J=8.7, 1.9 Hz, 1H), 8.06 (d, J=8.6Hz, 1H), 7.99 (d, J=2.5Hz, 1H), 7.67 (dd, J=8.5, 2,4Hz, 1H), 7.59 -7.52 (m, 2H), 7.49 (dd, J=8.6, 7.1Hz, 1H), 7.05 (dd, J=5.3, 3.3Hz, 2H), 6.95 (d, J=6.9Hz, 1H), 6.91 (d, J=9.4Hz, 1H), 6,52 (s, 1H), 4.98 (dd, J=12.7, 5.4Hz, 1H), 3.61 - 3.50 (m, 4H), 3.50 - 3.20 (m, 17H), 2.81 (ddd, J=16.9, 13.7, 5.4Hz, 1H), 2.74 - 2.66 (m, 1H), 2.65 - 2.57 (m, 1H), 2.55 - 2.51 (m, 1H), 2.51 - 2.46 (m, 2H), 2.34 – 2.24 (m, 1H), 2.00 - 1.88 (m, 1H). LCMS: m/z 1099.4 [M+1].

Supplementary Note 2: Synthetic Scheme of JH-XI-10-02 degrader

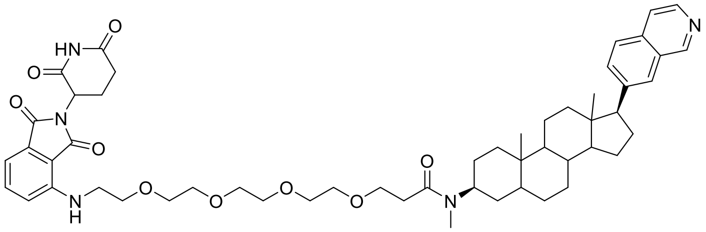

1-((2-(2,6-dioxopiperidin-3-yl)-1,3-dioxoisoindolin-4-yl)amino)-N-((3S,17S)-17-(isoquinolin-7-yl)-10,13-dimethylhexadecahydro-1H-cyclopenta[a]phenanthren-3-yl)-N-methyl-3,6,9,12-tetraoxapentadecan-15-amide

To a solution of (3S,8R,10S,13S,14S,17S)-17-(isoquinolin-7-yl)-N,10,13-trimethylhexadecahydro-1H-cyclopenta[a]phenanthren-3-amine (20 mg, 0.048 mmol) in DMF was added HATU (37 mg, 0.096 mmol) and 1-((2-(2,6-dioxopiperidin-3-yl)-1,3-dioxoisoindolin-4-yl)amino)-3,6,9,12-tetraoxapentadecan-15-oic acid (28 mg, 0.053 mmol) followed by DIEA (42 µL, 0.24 mmol). The reaction was stirred for 30 minutes and then injected directly onto the HPLC and purified using a gradient of 1-90% ACN in H_2O to give the title compound as a yellow solid (8 mg, 18% yield). ^{1}H NMR (500 MHz, DMSO): δ 11.09 (s, 1H), 9.71 (s, 1H), 8.60 (s, 1H), 8.36-8.29 (m, 3H), 8.19-8.16 (m, 1H), 7.99 (t, *J* = 5Hz, 1H), 7.60-7.54 (m, 1H), 7.15 (dd, *J* = 5Hz, 8Hz, 1H), 7.04 (d, *J* = 6Hz, 1H), 5.12- 5.03 (m, 1H), 4.27 (m, 1H), 3.64-3.59 (m, 4H), 3.54-3.45 (m, 14H), 2.99-2.84 (m, 2H), 2.79 (s, 3H), 2.06-1.95 (m, 3H), 1.83-0.91 (m, 24H), 0.77 (s, 3H), 0.43 (s, 3H). MS m/z: 921.49 $[M+H]^+$.

Supplementary References

1. Hardy, R. R. *et al.* Immunological Reviews B-cell commitment, development and selection. *Immunol. Rev.* **175**, 23–32 (2000).

2. Hardy, R. R. B-cell commitment: Deciding on the players. *Curr. Opin. Immunol.* **15**, 158–165 (2003).
