# Supplementary material for: A kinase-independent role for CDK8 in BCR-ABL1+ leukemia
Source: Nat Commun. 2019 Oct 18;10:4741. doi: 10.1038/s41467-019-12656-x (PMC6802219; doi:10.1038/s41467-019-12656-x)
Supplement: Supplementary file 2 — Reporting Summary [file 41467_2019_12656_MOESM2_ESM.pdf]

nature research

Corresponding author(s): Veronika Sexl
Last updated by author(s): Sep 4, 2019

Reporting Summary

Nature Research wishes to improve the reproducibility of the work that we publish. This form provides structure for consistency and transparency in reporting. For further information on Nature Research policies, see Authors & Referees and the Editorial Policy Checklist.

Statistics

For all statistical analyses, confirm that the following items are present in the figure legend, table legend, main text, or Methods section.

n/a	Confirmed	
☐	☒	The exact sample size (*n*) for each experimental group/condition, given as a discrete number and unit of measurement
☐	☒	A statement on whether measurements were taken from distinct samples or whether the same sample was measured repeatedly
☐	☒	The statistical test(s) used AND whether they are one- or two-sided<br>*Only common tests should be described solely by name; describe more complex techniques in the Methods section.*
☒	☐	A description of all covariates tested
☒	☐	A description of any assumptions or corrections, such as tests of normality and adjustment for multiple comparisons
☐	☒	A full description of the statistical parameters including central tendency (e.g. means) or other basic estimates (e.g. regression coefficient) AND variation (e.g. standard deviation) or associated estimates of uncertainty (e.g. confidence intervals)
☒	☐	For null hypothesis testing, the test statistic (e.g. *F*, *t*, *r*) with confidence intervals, effect sizes, degrees of freedom and *P* value noted<br>*Give P values as exact values whenever suitable.*
☒	☐	For Bayesian analysis, information on the choice of priors and Markov chain Monte Carlo settings
☒	☐	For hierarchical and complex designs, identification of the appropriate level for tests and full reporting of outcomes
☐	☒	Estimates of effect sizes (e.g. Cohen's *d*, Pearson's *r*), indicating how they were calculated

Our web collection on statistics for biologists contains articles on many of the points above.

Software and code

Policy information about availability of computer code

Data collection	*Provide a description of all commercial, open source and custom code used to collect the data in this study, specifying the version used OR state that no software was used.*
Data analysis	*Provide a description of all commercial, open source and custom code used to analyse the data in this study, specifying the version used OR state that no software was used.*

For manuscripts utilizing custom algorithms or software that are central to the research but not yet described in published literature, software must be made available to editors/reviewers. We strongly encourage code deposition in a community repository (e.g. GitHub). See the Nature Research guidelines for submitting code & software for further information.

Data

Policy information about availability of data

All manuscripts must include a data availability statement. This statement should provide the following information, where applicable:

- Accession codes, unique identifiers, or web links for publicly available datasets
- A list of figures that have associated raw data
- A description of any restrictions on data availability

The source data and uncropped gel pictures underlying Figs. 1a-e, 2a-f, h, i, 3a-i, 4b-d, f-i, 5a-b, 6b-d and 7d-j and supplementary Figs. 1a, 2a-f, 3a-i, 5a-b, 6b-c, 8a-d are provided as a source data file. Data that support the findings of this study are available from the authors upon reasonable request.

Field-specific reporting

Please select the one below that is the best fit for your research. If you are not sure, read the appropriate sections before making your selection.

☒ Life sciences ☐ Behavioural & social sciences ☐ Ecological, evolutionary & environmental sciences

For a reference copy of the document with all sections, see nature.com/documents/nr-reporting-summary-flat.pdf

Life sciences study design

All studies must disclose on these points even when the disclosure is negative.

Sample size

The power for the basic characterization of the hematopoeitic system was calculated as follows: first we checked the distribution of the samples by a D'Agostino-Pearson test. If the samples are distributed in a parametric way the statistical analysis was conducted with an unpaired t-test or ANOVA, in a non-parametric case we used the Kruskal-Wallis test or Mann Whitney U-Test. In previously conducted experiments we worked successfully with a group of 10 animals (2 groups á 5 animals) per experiment which was repeated at least 2 times. For in vivo transplant experiments the power was calculated as follows: statistical analysis of the time point at which first signs of disease occur was performed via a Log-Rank Test. In previously conducted experiments we worked successfully with a group of 10 animals (2 groups á 5 animals) or 15 animals (3 groups á 5 animals) per experiment which was repeated at least 2 times. Within 18 days 80% of the control animals diseased. In case that 60-70% of the experimental animals do not disease until then, we calculated a power of 90%.

Data exclusions

We performed a Grubbs' test to determine whether one of the values in the list is a significant outlier from the rest. If so, we excluded it from the analysis.

Replication

All replications were successful.

Randomization

At least three independent leukemic cell lines of a given genotype were injected into female and male NSG mice, at a preferably equal ratio. In addition, in cases of different ages of recipient mice (all in a range of 6-9 weeks), mice of individual litters were subjected to one experimental cohort in preferably equal ratios.

Blinding

Transplantation studies: no blinded studies as the experimenter had to note which mouse number received which cell line.

Reporting for specific materials, systems and methods

We require information from authors about some types of materials, experimental systems and methods used in many studies. Here, indicate whether each material, system or method listed is relevant to your study. If you are not sure if a list item applies to your research, read the appropriate section before selecting a response.

Materials & experimental systems

n/a	Involved in the study	
☐	☒	Antibodies
☐	☒	Eukaryotic cell lines
☒	☐	Palaeontology
☐	☒	Animals and other organisms
☒	☐	Human research participants
☒	☐	Clinical data

Methods

n/a	Involved in the study	
☒	☐	ChIP-seq
☐	☒	Flow cytometry
☒	☐	MRI-based neuroimaging

Antibodies

Antibodies used

Provided in the supplementary data section "Flow cytometry antibodies used in this study" and "Western blot antibodies used in this study", pages #5-7.

Validation

We checked citations and validation statements of each antibody on the manufacturer's website. Specificity was tested by using negative (like knockouts or unstimulated samples) and positive controls during the assays.

Eukaryotic cell lines

Policy information about cell lines

Cell line source(s)

Cell lines were purchased from the ATCC or were kindly provided by Peter Valent who purchased them from the Leibnitz Institute DSMZ-German Collection of Microorganisms and Cell Cultures.

Authentication

None of the used cell lines was authenticated in our lab.

Mycoplasma contamination	All cell lines were regularly tested negative for Mycoplasma contaminations
Commonly misidentified lines (See ICLAC register)	N/A

Animals and other organisms

Policy information about studies involving animals; ARRIVE guidelines recommended for reporting animal research

Laboratory animals	Conditional C57Bl/6N-Cdk8fl/fl (Cdk8tm1c(EUCOMM)Hmgu) were breed to B6N-Tg(Mx1Cre) and B6N-Tg(Vav-Cre). Cdk8fl/fl, Cdk8fl/flMx1Cre, Cdk8fl/flVav-Cre, Ly5.1+(CD45.1+), Ly5.1/2+ (CD45.1+ and CD45.2+) and NSG (NOD.Cg-PrkdcscidIl2rgtm1Wjl/SzJ; The Jackson Laboratory) were maintained under pathogen-free conditions at the University of Veterinary Medicine Vienna.
Wild animals	No wild animals are included.
Field-collected samples	No field-collected samples are included.
Ethics oversight	All animal experiments were approved by the institutional ethics committee and granted by the national authority (Austrian Federal Ministry of Science and Research) according to Section 8ff of Law for Animal Experiments under license BMWF-68.205/0218-II/3b/2012 and were conducted according to the guidelines of FELASA and ARRIVE.

Note that full information on the approval of the study protocol must also be provided in the manuscript.

Flow Cytometry

Plots

Confirm that:

- [x] The axis labels state the marker and fluorochrome used (e.g. CD4-FITC).
- [x] The axis scales are clearly visible. Include numbers along axes only for bottom left plot of group (a 'group' is an analysis of identical markers).
- [ ] All plots are contour plots with outliers or pseudocolor plots.
- [x] A numerical value for number of cells or percentage (with statistics) is provided.

Methodology

Sample preparation	Single cell suspensions of splenocytes, thymus and BM were prepared. For blood analysis the erythrocytes were lysed using BD FACS Lysing Solution according to manufacturer´s protocol (BD Bioscience).
Instrument	FACSCanto II BD Biosystems
Software	BD FACSDiva V8.0 and FlowJo V10 software
Cell population abundance	N/A
Gating strategy	Following stainings were gated according to publications: Hematopoietic stem cell staining (Wilson et al 2008 cell), Myeloid progenitor Staining (Herrera-Merchan, A. et al 2012 Nature Communications) and B-cell staining (Hardy et al. 2003 Curr. Opin. Immunol.) Simpler stainings like lineage, AnnexinV stainings or verification of GFP, dsRED, mCherry positive populations were gated positive like it is depicted in the Figure 3

- [ ] Tick this box to confirm that a figure exemplifying the gating strategy is provided in the Supplementary Information.
